# Digging into Lipid Membrane Permeation for Cardiac Ion Channel Blocker d-Sotalol with All-Atom Simulations

**DOI:** 10.3389/fphar.2018.00026

**Published:** 2018-02-01

**Authors:** Kevin R. DeMarco, Slava Bekker, Colleen E. Clancy, Sergei Y. Noskov, Igor Vorobyov

**Affiliations:** ^1^Department of Physiology and Membrane Biology, University of California, Davis, Davis, CA, United States; ^2^Department of Pharmacology, University of California, Davis, Davis, CA, United States; ^3^Biophysics Graduate Group, University of California, Davis, Davis, CA, United States; ^4^Hartnell College, Salinas, CA, United States; ^5^Centre for Molecular Simulations, Department of Biological Sciences, Faculty of Science, University of Calgary, Calgary, AB, Canada

**Keywords:** hERG, long QT syndrome, cardiotoxicity, CHARMM force field, molecular dynamics, umbrella sampling, lipophilicity, water-membrane partitioning

## Abstract

Interactions of drug molecules with lipid membranes play crucial role in their accessibility of cellular targets and can be an important predictor of their therapeutic and safety profiles. Very little is known about spatial localization of various drugs in the lipid bilayers, their active form (ionization state) or translocation rates and therefore potency to bind to different sites in membrane proteins. All-atom molecular simulations may help to map drug partitioning kinetics and thermodynamics, thus providing in-depth assessment of drug lipophilicity. As a proof of principle, we evaluated extensively lipid membrane partitioning of d-sotalol, well-known blocker of a cardiac potassium channel K_v_11.1 encoded by the hERG gene, with reported substantial proclivity for arrhythmogenesis. We developed the positively charged (cationic) and neutral d-sotalol models, compatible with the biomolecular CHARMM force field, and subjected them to all-atom molecular dynamics (MD) simulations of drug partitioning through hydrated lipid membranes, aiming to elucidate thermodynamics and kinetics of their translocation and thus putative propensities for hydrophobic and aqueous hERG access. We found that only a neutral form of d-sotalol accumulates in the membrane interior and can move across the bilayer within millisecond time scale, and can be relevant to a lipophilic channel access. The computed water-membrane partitioning coefficient for this form is in good agreement with experiment. There is a large energetic barrier for a cationic form of the drug, dominant in water, to cross the membrane, resulting in slow membrane translocation kinetics. However, this form of the drug can be important for an aqueous access pathway through the intracellular gate of hERG. This route will likely occur after a neutral form of a drug crosses the membrane and subsequently re-protonates. Our study serves to demonstrate a first step toward a framework for multi-scale *in silico* safety pharmacology, and identifies some of the challenges that lie therein.

## Introduction

The continuing failure to accurately predict the risk of drug toxicity is the primary reason for drug candidates being abandoned or approved drugs being removed from the market (Chi, 2013), illustrating the critical need for a more rational approach to drug development. One example of such a need is the longstanding failure of drug-based treatment of cardiac arrhythmias. The SWORD clinical trial (Waldo et al., 1996) famously showed that the antiarrhythmic drug d-sotalol, which we focus on in this work, actually *increased* mortality and risk of sudden cardiac death in patients, leading to its removal from the marketplace. Similarly, the gastrokinetic agent cisapride has been removed from the market in many countries due to its arrhythmogenic potential (Quigley, 2011), and a number of such cases for drugs and drug candidates with diverse pharmacological action has been growing over the years. Each year, over 360,000 people die in the US die from cardiac arrhythmias that are often drug-induced, demonstrating that the pharmacological assessment of cardiotoxicity still remains significantly hindered (Benjamin et al., 2017). The proposed Comprehensive *in vitro* Proarrhythmia Assay (CiPA) initiative is intended to address this shortcoming by improving predictions of pro-arrhythmic drug proclivities through the combination of *in vitro* assays on several cardiac ion channels and multi-scale modeling and simulation (Colatsky et al., 2016; Fermini et al., 2016). Atomistic MD simulations have the potential to serve as part of such *in silico* screen (Clancy et al., 2016) for the development of cardiac-safe medicines, and can be used to identify molecular determinants of acquired arrhythmogenesis.

On the molecular level, drug-induced arrhythmogenesis is typically associated with the binding of drugs to cardiac ion channels, membrane proteins responsible for the propagation of electrical signal in cardiomyocytes. It is known that multiple environmental factors, including drug blockade, can modulate the gating and permeation of many ion channels. More specifically, experimental studies aimed at understanding ion channel blockade by drugs often focus on mapping binding sites at or around the intra-cellular cavity of the ion channel. This assumes, either explicitly or implicitly, that a drug (often weakly cationic) is able to diffuse from the intra-cellular space and physically occlude ion permeation. Such a mechanism is supported, for example, by the role of two intra-cavity residues (F656 and Y652) in the drug-induced current block of the voltage gated potassium channel K_V_11.1 (also known as hERG), which is considered a major drug anti-target due to its promiscuous binding of many drug-like molecules (Vandenberg et al., 2012).

Many of the common ion channel blockers are weak bases with a pK_a_ of ~7.8–8.5. Thus, at a physiological pH of 7.4, up to ~7–28% of drug molecules remain uncharged, and therefore potentially capable of interacting with the channel by traversing a lipophilic pathway in the plasma membrane toward a binding site, either on the lipid-facing exterior of the channel or within the channel pore via passage through lipid-facing fenestrations. A possible lipophilic access route has been established for ivabradine blockade of hERG in a recent study that implicated a lipid-facing residue (M651) as critical for drug-induced blockade (Lees-Miller et al., 2015). This finding was further substantiated by the recent publication of Cryo-EM structures of hERG (putatively open), and related EAG (putatively closed) channels, suggesting that F656 and M651 can be exposed to lipids in either channel state (Whicher and MacKinnon, 2016; Wang and MacKinnon, 2017). Furthermore, hERG block by the endogenous components of cardiac membranes has also been well-established, with various lipophilic molecules including hormones (Yang et al., 2017), ceramides (Ganapathi et al., 2010; Sordillo et al., 2015), sphingosine-1-phosphate (Sordillo et al., 2015), and polyunsaturated fatty acids (Guizy et al., 2005; Moreno et al., 2012) blocking hERG but without obvious intra-cellular access to the intra-cavity site. Therefore, mapping the lipophilic pathways for common ion channel blockers and understanding the chemistry of drug-lipid interactions remains an unmet pharmacological challenge.

The complexity in understanding the lipophilic access pathways of many blockers arises from their chemical structure. Most drug molecules can coexist in multiple ionization states with different membrane permeabilities or localization on the bilayer surface and consequent access to binding sites in hERG. Hence, significant challenges exist in developing a framework for atomic-scale *in silico* screening and predictive pharmacology. One example is *the lack of robust topologies and parameters defined for most drugs in popular MD force fields*, necessitating their *de novo* development. This task requires computationally expensive calculations of quantum mechanical (QM) optimized molecular geometries and atomic charge distributions, and the time-consuming process of fitting molecular mechanical (MM) parameters to the optimal computed QM data. Here we have developed CHARMM generalized force field (CGENFF) (Vanommeslaeghe et al., 2010) parameters for the hERG blocker d-sotalol, which has high cardiotoxic risk (Colatsky et al., 2016) for the ventricular tachycardia characterized by Torsades de Pointes (TdP) arrhythmias (Waldo et al., 1996; Yap and Camm, 2003). Preliminary parameters for the intermediate-TdP risk compound cisapride (Colatsky et al., 2016), and low-risk compound moxifloxacin (Haverkamp et al., 2012) were developed for the purpose of comparing their membrane affinities, and will be briefly discussed as well.

Computing the free energy cost required for drugs to partition from bulk solution across the cell membrane represents a critical test for drug model viability used in MD simulations. This is because the membrane permeability of a drug not only determines its bioavailability, but is also linked to its medically relevant concentration, and pathway to its target. Many drugs are delivered to their targets via a lipophilic pathway, and drug permeation across lipid membranes is crucial for their absorption by tissue, metabolism, extraction from the body, and toxicity (ADME-Tox) (Yu and Adedoyin, 2003). This is especially relevant for predicting propensity for off-target effects of a drug, which is necessarily linked to its tissue permeability. Empirically derived ADME-Tox drug profiles, however, are inherently limited, lacking transferability to different drug classes, and providing no information regarding the structural determinants of membrane-drug distribution or kinetics (Swift and Amaro, 2013). Obtaining these measurements through MD simulation represents a final major challenge: namely, *obtaining sufficient sampling of the configurational space in a modeled system to calculate accurate thermodynamic quantities of interest*. Ideally, unbiased all-atom MD simulations of drug permeation across large, explicit lipid membranes would provide the most accurate kinetic and thermodynamic profiles for membrane-drug interactions (Swift and Amaro, 2013), however the sampling (or simulation time) mandated by such an exhaustive approach makes it computationally prohibitive. Fortunately, more computationally tractable techniques for enhanced sampling exist that allow for the robust calculation of membrane distribution coefficients and permeability measurements of an isolated drug across a small membrane patch. We have employed one such technique, umbrella sampling (US) (Torrie and Valleau, 1977), in this report in order to compute the free energies and diffusion coefficients required for drugs to pass through the cell membrane. Similar approaches have been used for various drug molecules in a number of other studies (Carpenter et al., 2014; Di Meo et al., 2016; Bennion et al., 2017), including previous works by our groups (Boiteux et al., 2014; Yang et al., 2016). The approaches and data presented here serve as preliminary steps in overcoming the many challenges that arise in the messy task of atomistic *in silico* predictive cardiovascular pharmacology.

## Materials and methods

### Drug force field parameterization

We obtained starting molecular structures from either PubChem (Kim et al., 2016) (CID 5253 for d-sotalol) or the ZINC (Irwin and Shoichet, 2005) (3775140 for cisapride, 3826253 for moxifloxacin) databases, and used them to generate initial guesses for partial atomic charges and other force field parameters (i.e., bond lengths, bond angles, dihedral angles) using CGENFF program, version 1.0 (Vanommeslaeghe and MacKerell, 2012; Vanommeslaeghe et al., 2012).

Initial topology and parameters for SotC and SotN, were subsequently validated and optimized using QM target data following the suggested CGENFF force field methodology (Vanommeslaeghe et al., 2010). High-quality parameters not already present in CGENFF are assigned from existing parameters based on chemical analogy, with poor chemical analogy corresponding to a high penalty score for use in MD simulation (Vanommeslaeghe et al., 2012). Our optimizations focused on such high-penalty, poorly analogous parameters generated by the CGENFF program. Quantum mechanical (QM) target data for parameter optimization were obtained utilizing Møller–Plesset (MP2) and Hartree-Fock (HF) electronic structure methods and the 6–31(d) basis set using the Gaussian 09 program (Frisch et al., 2009).

MP2/6-31G(d) molecular dipole magnitude and orientation as well as scaled HF/6-31G(d) interaction energies with water were used for partial atomic charge optimization for compatibility with the CHARMM atomistic biomolecular force fields (MacKerell, 2004). The gas-phase MP2/6-31G(d) dipole, along with HF/6-31G(d) interaction energies, should be overestimated by CHARMM (by ~16% for the latter) in order to account for polarization in aqueous media (MacKerell, 2004; Vanommeslaeghe et al., 2010). Internal bond and angle parameters were validated or modified based on comparison of MP2/6-31G(d) and CHARMM optimized geometries and scaled vibrational frequencies. For bond lengths and angles, respective differences within 0.01 Å and 1° between QM and CHARMM values were sought. Dihedral angle parameters were optimized to reproduce MP2/6-31G(d) potential energy scans for rotation around a particular bond. We used the Force Field Toolkit plugin (fftk) (Mayne et al., 2013) for the Visual Molecular Dynamics program (VMD) (Humphrey et al., 1996) in order to generate files for QM reference calculations and to perform parameter optimizations. We were able to achieve substantial improvement over the initial CGENFF generated parameters (highlighted in **Figure 3C** for a selected dihedral angle energy profile), with markedly better agreement between CHARMM and QM geometries, vibrational frequencies, and interactions with water. Final topology and parameters for SotC and SotN are provided in the Supplementary Information. Optimized parameters for charged cisapride and zwitterionic moxifloxacin, obtained using the same methodology, will be subsequently published after additional validation and any necessary improvement.

### Drug membrane partitioning: molecular systems

Partitioning of charged (SotC) and neutral d-sotalol (SotN), charged cisapride (CisC), and a zwitterionic form of moxifloxacin (MoxZ) were assessed using CHARMM (Brooks et al., 1983, 2009) and NAMD (Phillips et al., 2005) programs. CHARMM-GUI tool (Jo et al., 2008) was used in order to build the simulation systems, which consisted of 128 1-palmitoyl-2-oleoylphosphatidylcholine (POPC) lipids, ~7,000 water molecules, 21 or 22 K^+^ and 22 Cl^−^ ions to ensure 0.15 M electrolyte concentration and overall electrical neutrality, and one drug molecule, totaling ~38,250 atoms.

A separate set of simulations that investigated membrane composition was equilibrated with NAMD and run on Anton 2 supercomputer (Shaw et al., 2014). In these simulations lipid membranes were composed of either pure POPC or a mixture of 85% POPC and 15% of 1-palmitoyl-2-oleoylphosphatidylserine (POPS) lipids. These systems were larger and contained ~103,000 atoms with 256 lipids, 15 SotC or 16 SotN molecules, ~22,800 water molecules, 50–88 K^+^ and 50–65 Cl^−^ ions.

CHARMM biomolecular, and compatible CGENFF forcefields were used throughout all simulations. In particular, C36 lipid (Klauda et al., 2010) and standard CHARMM ion parameters (Beglov and Roux, 1994), newly developed CGENFF drug parameters (see above) along with the TIP3P water model (Jorgensen et al., 1983) were utilized.

### Drug membrane partitioning: molecular dynamics simulations

CHARMM simulations of SotC, SotN, CisC, and MoxZ, and NAMD simulations of SotC and SotN in a hydrated 128 lipid POPC membrane were carried out in *NPT* ensemble with 1 atm of pressure maintained by Langevin piston barostat (Feller et al., 1995) and 310 K temperature controlled by Nosé-Hoover thermostat (Nosé, 1984; Hoover, 1985). Tetragonal cells with periodic boundary conditions (PBC) were used in all the simulations, with P2_1_ space group (Dolan et al., 2002) utilized in CHARMM runs. SHAKE algorithm (Ryckaert et al., 1977) was employed to fix the bonds to all hydrogen atoms, allowing a time step of 2 fs for all our simulations. Electrostatic interactions were computed via Particle Mesh Ewald (Darden et al., 1993), with a mesh grid of 1 Å.

For partitioning calculations of each drug we used the US method (Torrie and Valleau, 1977) with 81 independent simulation windows, placing the center of mass (COM) of the drug in 1 Å intervals from −40 Å to 40 Å with respect to COM of the membrane. The COM of the drug was restrained along the *z* axis with a force constant of 2.5 kcal/mol/Å^2^ to provide sufficient sampling with an additional 5 kcal/mol/Å^2^ cylindrical constraint applied to prevent the drift of the molecule in the *xy* plane (Li et al., 2008). Free energy or potential of mean force (PMF) profiles was computed using weighted histogram analysis method (WHAM) (Kumar et al., 1992).

SotC and SotN simulations ran for 15 ns with NAMD and 10 ns with CHARMM per window. To improve sampling, for NAMD runs we used additional US windows from −20 Å to 20 Å, whereas 7 central windows (i.e., for |*z*| ≤ 3 Å) were used for CHARMM SotC simulations, all running for the same simulation time as the original runs (see Supplementary text). Based on solvation analysis of SotC and SotN (Figure S5), we discarded the first 4 ns to account for equilibration. For consistency, similar procedure was followed for CHARMM simulations of CisC and MoxZ, both of which ran 10 ns/window plus additional 10 ns for the 5 central windows (|*z*| ≤ 2 Å) of CisC.

Unbiased MD simulations were run for larger membrane systems with several SotC or SotN molecules. First, systems were equilibrated for 50 ns using NAMD and the simulation parameters mentioned above. Then, production simulations were run for 500 or 1000 ns (for SotN system with POPC/POPS mixed membrane) using Anton 2 software (Shaw et al., 2014) version 1.31.0. These simulations were carried out using tetragonal PBC in the *NPT* ensemble at 310 K, a 2 fs time step with non-bonded long range interactions computed every 6 fs using the RESPA multiple time step algorithm (Tuckerman et al., 1992). The multi-integrator (multigrator) algorithm (Lippert et al., 2013) was used for temperature and semi-isotropic pressure coupling, whereas a novel u-series method (Shaw et al., 2014) was used for handling long-range electrostatic interactions. An electric field in the *z* direction was applied, gradually increasing from 0 to 160 mV during the first 100 ns of the simulation. A long-range Lennard-Jones (LJ) correction (beyond cutoff) was not used as was suggested for C36 lipid force field (Klauda et al., 2010).

### Drug membrane partitioning: simulation analyses

Solvation numbers were computed as number of oxygen atoms of water, lipid phosphate or ester functional groups within 4.25 Å of drug non-hydrogen atoms, with this distance cutoff obtained from an analysis of corresponding radial distribution functions (see Figure S6). Drug orientation was computed based on a polar angle θ between *z* axis corresponding to a bilayer normal and drug N1^…^S vector, which is almost anti-parallel to its dipole orientation (see **Figure 3**). Average angles were computed as:

(1) $$\begin{array}{l} <\theta_{\text{N}1\ldots \text{S}}>=\text{atan}2\left(<\text{sin}\theta >,<\text{cos}\theta >\right) \end{array}$$

whereas corresponding order parameters were computed as (Vorobyov et al., 2012)

(2) $$\begin{array}{l} {\text{S}}_{\text{N}1\ldots \text{S}}=½\left(3<{\text{cos}}^{2}\theta >-1\right) \end{array}$$

Drug water-membrane partition coefficients were calculated as (Vorobyov et al., 2012):

(3) $$\begin{array}{l} \text{K}\left(\text{wat}\to \text{mem}\right)=\frac{1}{z_{2}-z_{1}}\overset{z_{2}}{\underset{z_{1}}{\int }}e^{-\frac{\left\{W\left(z\right)-W\left(z_{1}\right)\right\}}{k_{B}T}}dz \end{array}$$

where *W*(*z*) is the PMF, *z*_1_ and *z*_2_ are points in aqueous solution on opposite sides of the membrane, *k*_B_ is Boltzmann constant, and *T* is the absolute temperature. Partitioning free energies were calculated as

(4) $$\begin{array}{l} \Delta G\left(\text{wat}\to \text{mem}\right)={–\text{k}}_{\text{B}}\text{T ln K}\left(\text{wat}\to \text{mem}\right) \end{array}$$

Error bars were estimated from PMFs by propagation of uncertainties.

To estimate the 1D diffusion constant in the *z* direction, *D*(*z*_*i*_), we analyzed the corresponding US windows with Hummer's method (Hummer, 2005):

(5) $$\begin{array}{l} D\left(z_{i}\right)=\frac{{\langle \delta z^{2}\rangle }_{i}}{\tau_{i}} \end{array}$$

where ${\langle \delta z^{2}\rangle }_{i}$ and τ_*i*_ are the mean square deviation from the average position and the position correlation time for US window *i*.

(6) $$\begin{array}{l} \tau_{i}=\underset{s\to 0}{\lim}\tau_{i}(s)=\underset{s\to 0}{\lim}\frac{{\hat{C}}_{z}(s;z_{i})}{{\langle \delta z^{2}\rangle }_{i}}=\underset{s\to 0}{\lim}\frac{\int_{0}^{\infty }e^{-st}{\langle \delta z(t)\delta z(0)\rangle }_{i}\text{d}t}{{\langle \delta z^{2}\rangle }_{i}} \end{array}$$

Ĉ_*z*_(*s*; *z*_*i*_) is the Laplace transform of the position autocorrelation function *C*_*z*_(*t*; *z*_*i*_):

(7) $$\begin{array}{l} {Ĉ}_{z}\left(s;z_{i}\right)=\int_{0}^{\infty }e^{-st}C_{z}\left(t;z_{i}\right)\text{d}t \end{array}$$

where *C*_*z*_(*t*; *z*_*i*_) = 〈δ*z*(*t*)δ*z*(0)〉_*i*_, *s* is the inverse time and δ*z* = *z*−〈*z*〉_*i*_ is the drug COM position displacement.

Values of τ_*i*_(*s*) were calculated at *s*-values 0.01, 0.02, …, 0.1, 0.2, …, 1.0, 2.0, …, 10.0 ps^−1^. τ_*i*_(*s*) were extrapolated to *s* = 0 by fitting the function *a*/(*s*+*b*), where *a* and *b* are parameters, in the *s* range from 0.02 to 1.00 ps^−1^. See our previous study (Vorobyov et al., 2014) for more details.

Based on PMF and diffusion coefficient profiles we computed water-membrane drug permeability rate as,

(8) $$\begin{array}{l} P={\left(\int_{-L/2}^{L/2}\frac{\exp\left(W_{z}/k_{B}T\right)}{D\left(z\right)}\text{d}z\right)}^{-1} \end{array}$$

an integral over the local bilayer resistance (Marrink and Berendsen, 1994), spanning −14 ≤ *z* ≤ 14 Å for SotN and −20 ≤ *z* ≤ 20 Å for SotC (with PMF-values adjusted to be 0 at the borders), where the drug is expected to cross a central barrier; essential for modeling permeation via a single molecule PMF (Roux and Karplus, 1991). This description assumes we are in the diffusion limit, where the mean velocity is proportional to the mean force, which is valid if the drug displacement correlation length is short compared to the spatial variations in the force (Marrink and Berendsen, 1994).

## Results

### Comparative ionized drug membrane partitioning

First, we studied membrane partitioning of SotC and compared it to the partitioning of CisC and MoxZ, each drug form representing the dominant protonation state in aqueous solution at the physiological pH. We studied their translocation across POPC membranes using US MD simulations, which allow for more efficient sampling of energetically unfavorable drug distributions across a lipid membrane compared to conventional unbiased MD simulations. US works by restraining drug positions at different values of *z* across the membrane using a harmonic potential. Thus, we can compute free energy for drug positions along the bilayer normal, with *z* = 0 corresponding to membrane center.

**Figure 1:**
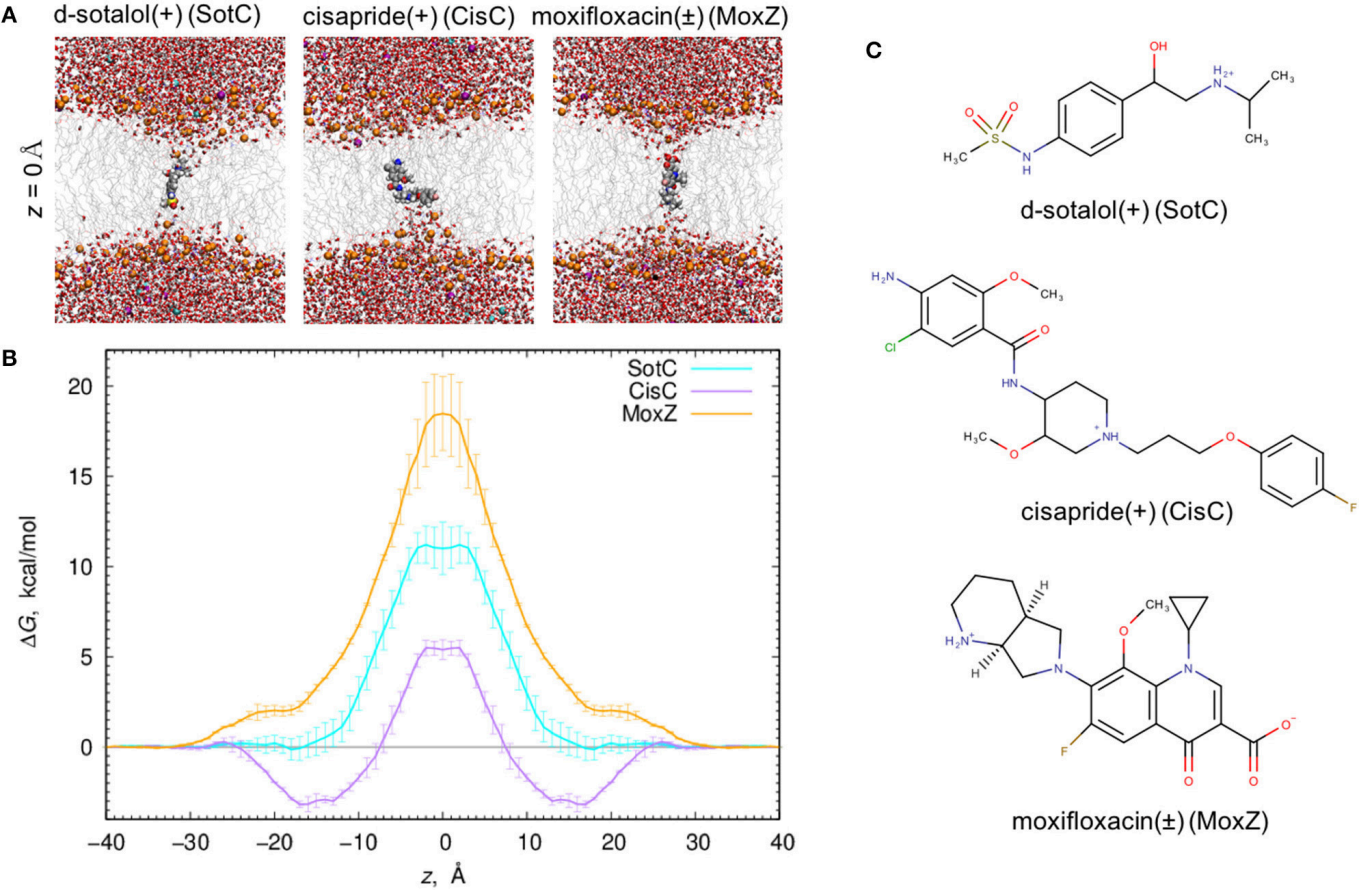
Ionized drug translocation across a POPC membrane. **(A)** Representative snapshots from a central (*z* = 0) umbrella sampling windows for cationic d-sotalol (SotC), cationic cisapride (CisC), and zwitterionic moxifloxacin (MoxZ). Drug molecules along with lipid P atoms (orange), K^+^ (purple) and Cl^−^ (cyan) ions are shown in a space-filling representation. Other elements are colored as follows: C—gray, H—white, O—red, N—blue, S—yellow. Water molecules are shown as tubes and lipid tails as wireframes. **(B)** PMF profiles for POPC membrane crossing for 3 drugs shown in **(A)**. Error bars represent measures of asymmetry. **(C)** Chemical structures of those drugs drawn using MarvinSketch program.

When all 3 drugs are located near *z* = 0 (see Figure 1A), we observed substantial membrane deformations, where they are coordinated by water molecules and lipid headgroups from one (for CisC) or both (for SotC and especially for MoxZ) membrane interfaces. Not surprisingly, such membrane deformations lead to substantial energetic penalties for ionized drugs to move across the membrane with the peak values at *z* = 0: around 18 kcal/mol for MoxZ, 10 kcal/mol for SotC and just 5 kcal/mol for CisC. Interestingly, such differences in peak free energy values correlate with computed MM drug dipole moments, which are 41.3 Debye for MoxZ, 15.5 Debye for SotC and 6.8 Debye for CisC for the same drug molecule “standard” positions and orientation (as defined by Gaussian software). For MoxZ, extensive membrane deformation exhibited by both leaflets are due to the positively charged ammonium and negatively charged carboxylate moieties at opposite ends of the molecule (Figure 1C). For SotC, a cationic secondary ammonium and polar sulfonamide groups can also attract water molecules or lipid headgroups. Both SotC and MoxZ can stretch along the membrane normal to interact with both bilayer interfaces. However, the situation is different for CisC, which also has several polar functional groups and a positively charged tertiary ammonium functionality at the center of the molecule, but it is floppier than those drugs and seems to be attracted to one membrane interface (see Figure 1). Also, CisC has a pronounced binding trough of around −3 kcal/mol at 14 ≤ |*z*| ≤ 17 Å. This suggests, that unlike SotC and MoxZ it will accumulate at water—membrane interface. The presence of the binding trough will also inadvertently increase a barrier a drug will need to overcome to cross a membrane from ~5 to 8 kcal/mol (see Figure 1B). These calculations suggest fairly high but surprisingly different energetic costs to cross the membrane for this collection of ionized molecules.

### Models of d-sotalol

**Figure 2:**
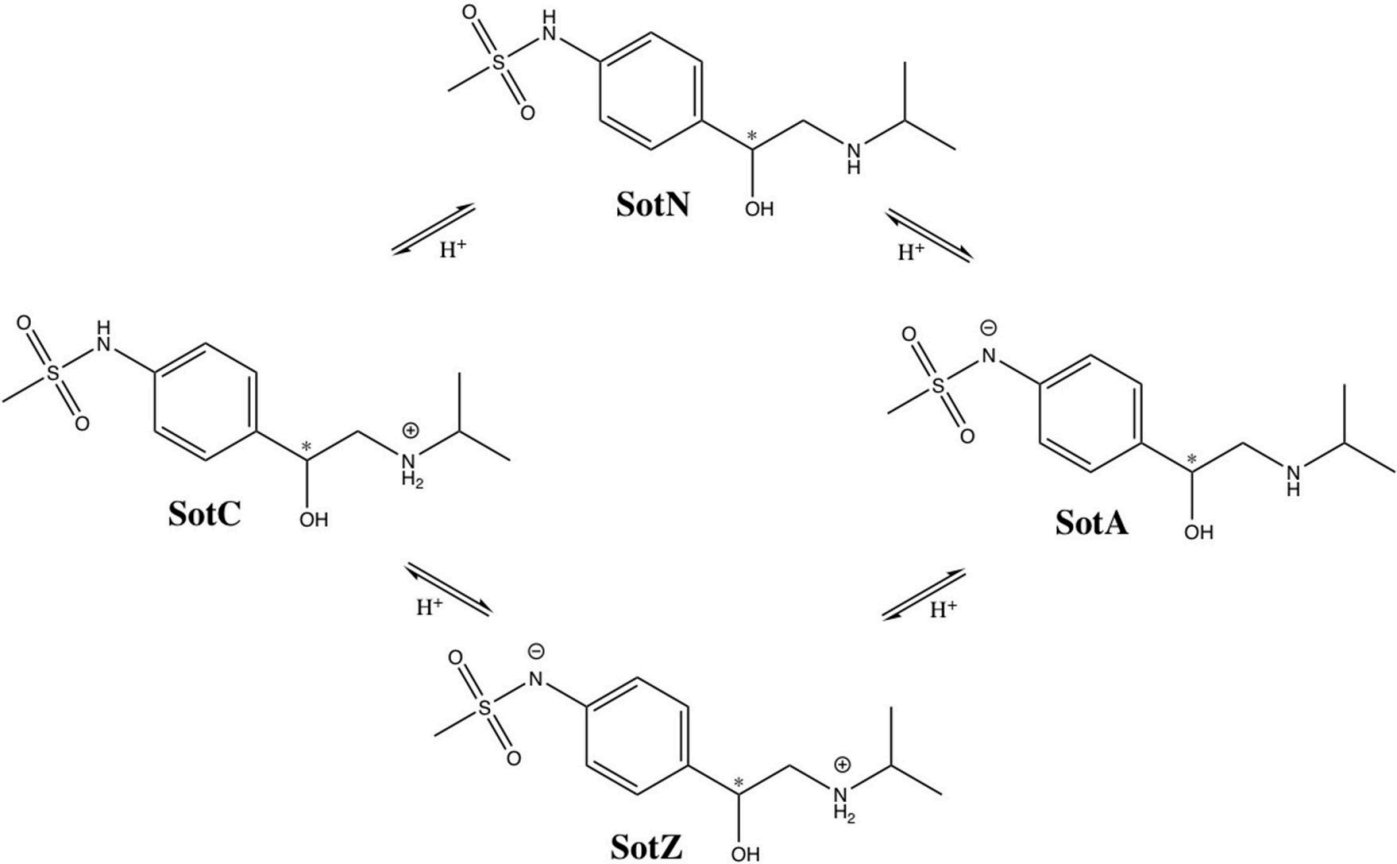
Protonation states of d-sotalol. Chemical structures were drawn using ChemDraw program. Asterisk (^*^) indicates chiral C atom.

We performed a more detailed analysis of different protonation states of d-sotalol, focusing on the energetics of its membrane crossing. Like many other drugs in aqueous solution, d-sotalol can exist in several protonation states depending on solution pH and other factors, such as proximity to specific protein residues. Data from the literature indicate that aqueous p*K*_a_-values for d-sotalol are 8.3 and 9.8 attributed to deprotonation of sulfonamide and secondary ammonium functionalities, respectively (Foster and Carr, 1992; Hancu et al., 2014). This indicates that at physiological pH 7.4, SotC is the predominant form (around 89%), while deprotonation of the sulfonamide functionality leads to a second dominant SotZ form (around 11%). At more basic pH, the secondary ammonium functionality will deprotonate as well, leading to a negatively charged, anionic form SotA (Figure 2).

However, there is yet another possibility, in which deprotonation of secondary ammonium group occurs first, leading to a neutral d-sotalol form (SotN). In fact, there is likely an equilibrium, and possibly interconversion, between SotN and SotZ forms, in which either one is favored depending on the polarity of the surrounding medium. We expect that a substantially less polar SotN form would be favored in the hydrophobic environment of the lipid membrane interior based on our MoxZ simulations discussed above, whereas a more polar SotZ might be favored in aqueous solution. Unfortunately, there are no experimental data to address this issue for d-sotalol. We performed a series of implicit solvent QM calculations, which seem to indicate slight preference for SotN even in bulk water (see Supplementary text for more information), but their accuracy is very uncertain. However, a recent experimental study using a combination of potentiometric titration and spectrophotometry measurements has suggested around 90% of zwitterionic and 10% of neutral form of moxifloxacin is present at physiological pH range, and that only a neutral form contributes to drug partitioning into a non-polar environment of lipid membranes or 1-octanol often used as a membrane mimetic (Langlois et al., 2005). This suggests that a neutral form of a drug is likely the one to undergo an unassisted membrane translocation.

**Figure 3:**
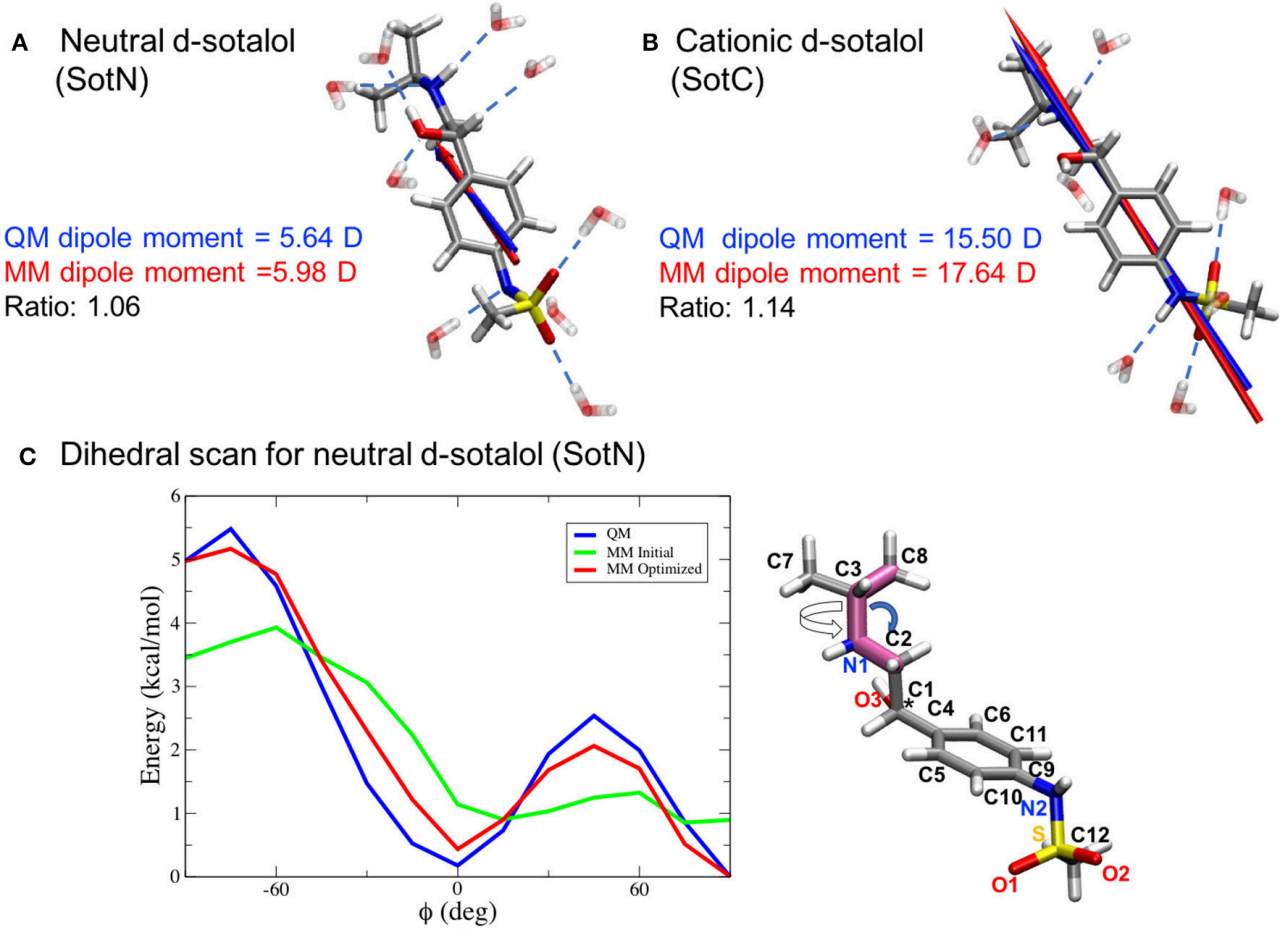
CHARMM force field parameter optimization of d-sotalol. The QM (blue arrow) and MM (red arrow) dipole moments for neutral, SotN **(A)**, and charged, SotC **(B)**, forms of d-sotalol are compared, and their QM optimized water interactions are shown by dashed blue lines. A sample dihedral angle C8-C3-N1-C2 optimization (bonds are highlighted in purple on SotN molecule) is shown in **(C)**, with reference QM computed energy scan in blue, non-optimized CGENFF energy scan in green, and optimized MM energy scan in red, demonstrating marked improvement. Asterisk (^*^) indicates chiral C atom (C1).

Since we are particularly interested in lipophilic access of cardiotoxic drugs known to block hERG, we have developed standard CHARMM (Klauda et al., 2010) compatible models of d-sotalol in charged (SotC) and neutral (SotN) forms. The QM and MM dipole moments for those d-sotalol forms and drug—water interactions probed for the model optimizations are shown in Figures 3A,B for SotN and SotC, respectively. Optimized CHARMM charges (Table S3) provide good agreement with QM target dipole values. The optimized MM dipole moments point in same direction (<1° difference in angle between QM and MM for both SotC and SotN) and are each within 20% difference in magnitude (SotN 6%, and SotC 14%). The water interaction distances were all within 0.4 Å of QM target values (see Tables S4, S5). The dipole moment is significantly higher for SotC (17.64 Debye), than for SotN (5.98 Debye), as is to be expected for charged vs. neutral species and in agreement with QM-values. Interaction energies with water were also in good agreement with QM-values with root mean square (RMS) and maximum errors of 0.8 and 1.5 kcal/mol for SotN (Table S5) as well as 1.6 and 3.0 kcal/mol (see Table S4) for SotC, respectively. No internal (bond, angle, dihedral angle) parameters needed to be optimized for SotC, whereas for SotN there was a high penalty score for the C2-N1-C3 bond angle (shown by blue arrow in Figure 3C), and optimization yielded a difference of 0.86° (i.e., <1° as required) between MM and QM values. Also for SotN, 7 dihedral angle parameter optimizations yielded marked improvement over CGENFF initial guesses (illustrated in Figure 3C for SotN C8-C3-N1-C2 dihedral angle highlighted in pink, with all the dihedral scan profiles shown in Figure S2), with optimized torsional energy minima within ~0.5 kcal/mol of QM values. For comparison, raw CGENFF dihedral parameters with high penalties yielded QM energy minima differences sometimes as high ~2 kcal/mol. These optimized parameters represent a significant improvement over initial guesses and should yield more accurate computed energetics from MD simulations.

At this time, we were not able to develop empirical models of the SotZ and SotA forms of the drug (Figure 2), since a negatively charged sulfonamide nitrogen atom type does not exist in either CHARMM biomolecular, or generalized (CGENFF) force fields. The fraction of these forms in aqueous solution or other media is uncertain, but based on a very high free energy barrier for zwitterionic moxifloxacin translocation (Figure 1 and discussion above) as well as the very large dipole moments for SotZ and SotA (see Table S1 and Supplementary text), we do not expect them to contribute substantially to the passive diffusion of d-sotalol across a lipid membrane, or the lipophilic access of this drug to hERG or other protein targets.

We should also mention that sotalol has a chiral center at C1 atom (shown by an asterisk in Figures 2, 3C), and in this study we only focused on S-enantiomer, d-sotalol. However, the developed force field parameters can be also used for R-enantiomer, l-sotalol, which will be also considered in our subsequent studies.

### d-Sotalol solvation and orientation across the membrane

We used our SotC and SotN models to investigate their interactions with a lipid membrane as they move across using US MD simulations. For those simulations we applied extensive sampling, especially important for hindered drug reorientation in the membrane interior (see Supplementary text for more information). We also performed those simulations with two popular biomolecular modeling packages, NAMD and CHARMM, with the former being more computationally efficient on our GPU (Graphics Processing Unit) cluster. However, CHARMM allows using P2_1_ symmetry to take into account likely changes in the areas of top and bottom bilayer leaflets as a drug moves through the membrane by shuffling lipid molecules between them as it happens. We established that the lipid membrane properties of our simulated systems are in agreement with experimental data in this case (See Supplemental text).

**Figure 4:**
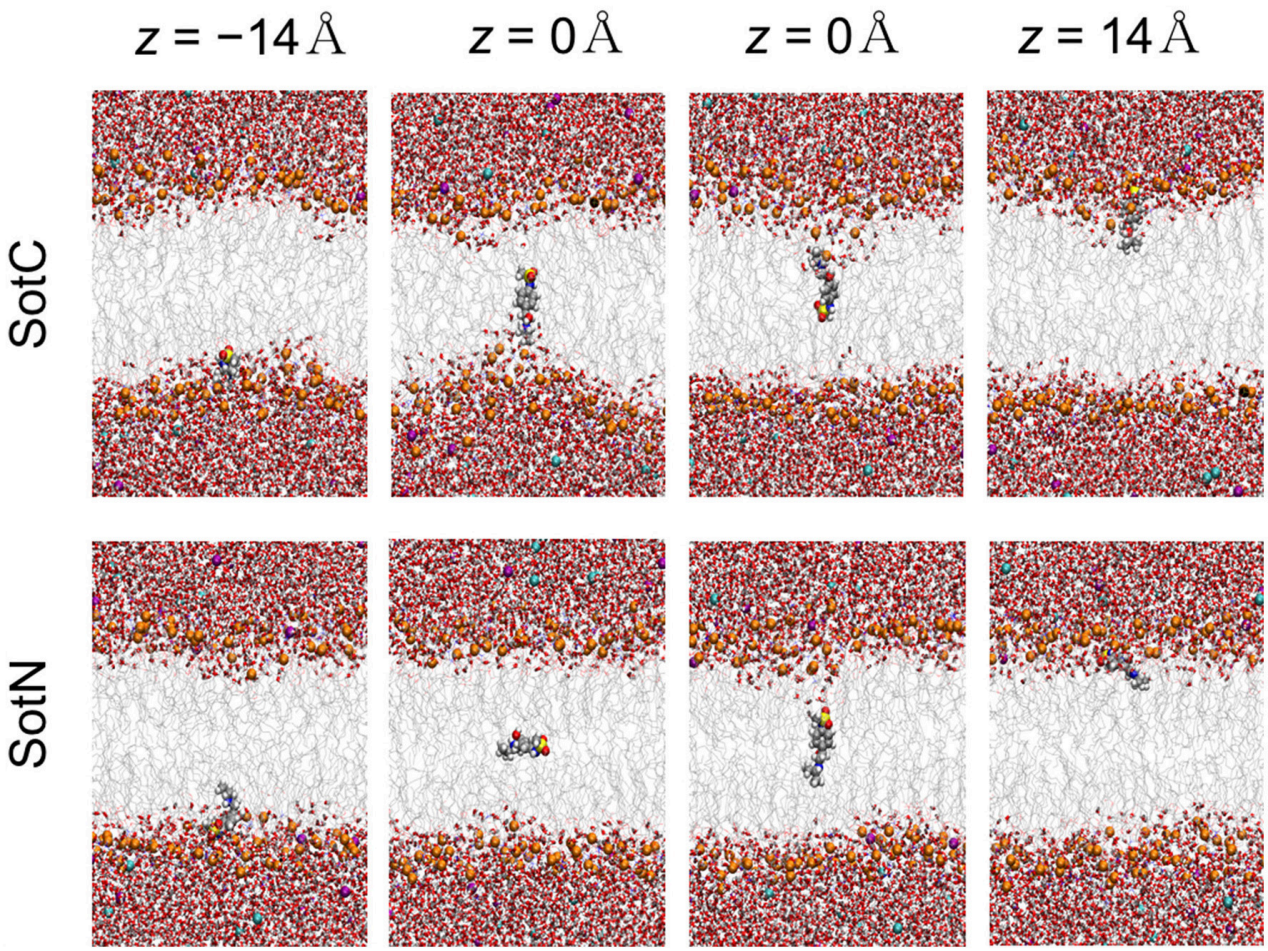
Representative snapshots of charged (SotC) and neutral (SotN) d-sotalol moving across a POPC membrane from umbrella sampling MD simulations. Reference d-sotalol center of mass (COM) *z* positions with respect to membrane COM are shown on the top. See Figure 1 caption for molecular representation and coloring information. Two structures for *z* = 0 for each drug represent final system snapshots from two independent simulations with a different initial drug orientation (see Supplementary text for more information).

We then started to investigate membrane—drug interactions, first, by looking at equilibrated system snapshots at the membrane center (*z* = 0 Å) and water/membrane interfacial region |*z*| = 14 Å, corresponding to free energy minimum for SotN (see Figure 4). It can clearly be seen that both charged and neutral drug molecules can adapt different orientations with respect to the membrane normal and can be solvated by both water molecules and lipid head groups even deep in the membrane interior for SotC in agreement with our CHARMM multiple-drug simulations shown in Figure 1 and discussed above. Interestingly, that in NAMD simulation snapshots shown in Figure 4, we observed that SotC while held around membrane center (*z* = 0) can adopt different long-lasting (see below) orientations “grabbing” water molecules and lipid head groups from either top or bottom membrane interface, but did not observe them making interfacial connections to both leaflets, as was observed in our CHARMM simulations (Figure 1).

**Figure 5:**
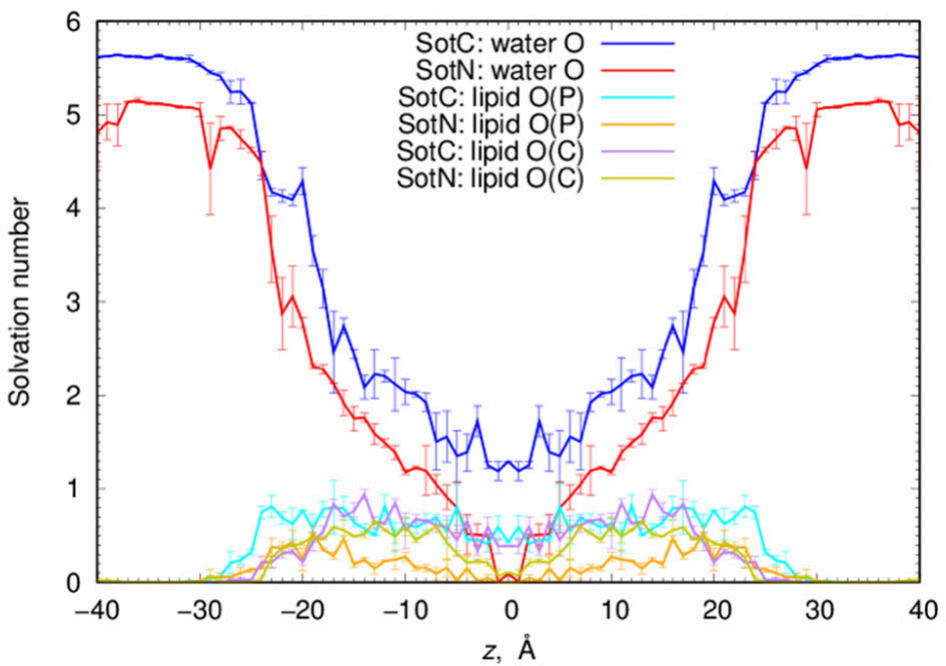
Analysis of d-sotalol solvation from umbrella sampling MD simulations. Solvation numbers of water or lipid oxygen atoms within 4.25 Å cutoff distance from non-hydrogen atoms of SotC or SotN were computed based on integrated radial distribution function (RDF) profiles. See Figure S6 for a few representative RDF profiles. Error bars shown in all the graphs are computed from profile asymmetries.

Next, we performed a quantitative analysis of drug solvation shown in Figure 5. While SotC and SotN are found in bulk water regions, for |*z*| > 25 Å (~5 Å beyond phosphate groups), they are solvated by ~5.5 and 5 water molecules, respectively. We defined the interfacial region as 15 < |*z*| < 25 Å, where 15 Å boundary was established based on an experimentally determined POPC hydrophobic thickness of 28.8 ± 0.6 Å (Kucerka et al., 2011). The water coordination remains the same as in bulk, until the drug reaches inside the core of the membrane, where we observe a bigger drop in the number of water molecules solvating SotN. In the center of the bilayer, at *z* = 0 Å, almost no water molecules are found coordinating the neutral drug, while at least 1.2 water molecules continue to coordinate the charged species. Additionally, when SotC is found at the interface or the hydrophobic core of the membrane, it is coordinated by lipid phosphate and carbonyl groups, while SotN remains virtually uncoordinated by these functional groups in the membrane core and has a similar coordination by carbonyl O and smaller by phosphate O atoms in the interfacial region (Figure 5).

**Figure 6:**
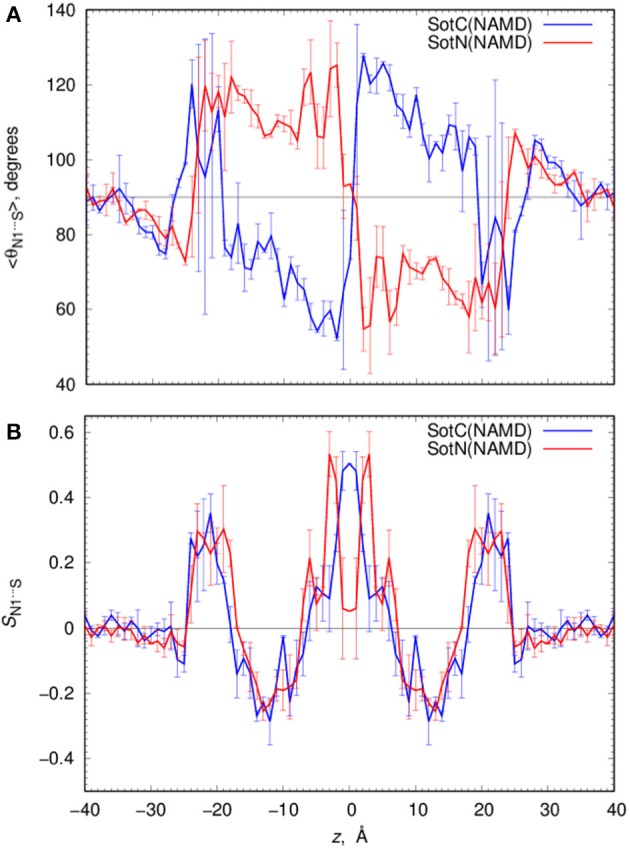
Analysis of d-sotalol tumbling during umbrella sampling MD simulations. **(A)** Average polar angle θ distribution for N1^…^S d-sotalol vector with respect to the *z* axis for charged (SotC, blue) and neutral (SotN, red) drug moving across POPC membrane. **(B)** Corresponding order parameter profiles for this vector with respect to the *z* axis. Error bars shown in all the graphs are computed from profile asymmetries. See Figures S7, S8 for a few representative θ(N1^…^S) time series.

Such solvation results in the preferential orientation of both SotC and SotN with respect to bilayer normal (coinciding with the *z* axis) as shown in Figure 6. There is no preferred orientation of both drugs in bulk water as expected, which is exemplified by average θ being around 90° and order parameter being 0 (see Figure 6 and top right panels in Figures S7, S8 for time series). There is a strong preference for N1^…^S vector of both drugs to be aligned with the z axis in the outer interfacial region i.e., at 20 < |*z*| < 25 Å, whereas there is some tendency for drugs to lie perpendicular to the membrane normal i.e., in the membrane plane (with order parameter *S* < 0) in the inner interfacial and outer core regions at 10 < |*z*| < 20 Å (see Figures S7, S8 for time series). In the inner core region (|z| < 10 Å) the drugs again become aligned or anti-aligned with the *z*-axis. Interestingly, the orientation of SotN and SotC in the inner interfacial and core regions seem to be opposite—with SotC favoring parallel orientation and SotN—antiparallel with the membrane normal for the drug positions with the negative *z*-values (Figure 6). This results from different relative affinities of SotC and SotN functional groups: the cationic ammonium group in SotC strongly attracts water molecules and lipid head groups, whereas its deprotonation makes its sulfonamide functionality a better attractor leading to this functional group re-orientation to be closer to the membrane interface. These interactions lead to hindered rotation (see Figures S7, S8) on the time scale of MD simulations we performed here (10–15 ns for each drug *z*) leading to difficulties sampling thermodynamics of drug—membrane interactions discussed below (see Supplemental text for more details).

### d-Sotalol energetics and protonation across the membrane

**Figure 7:**
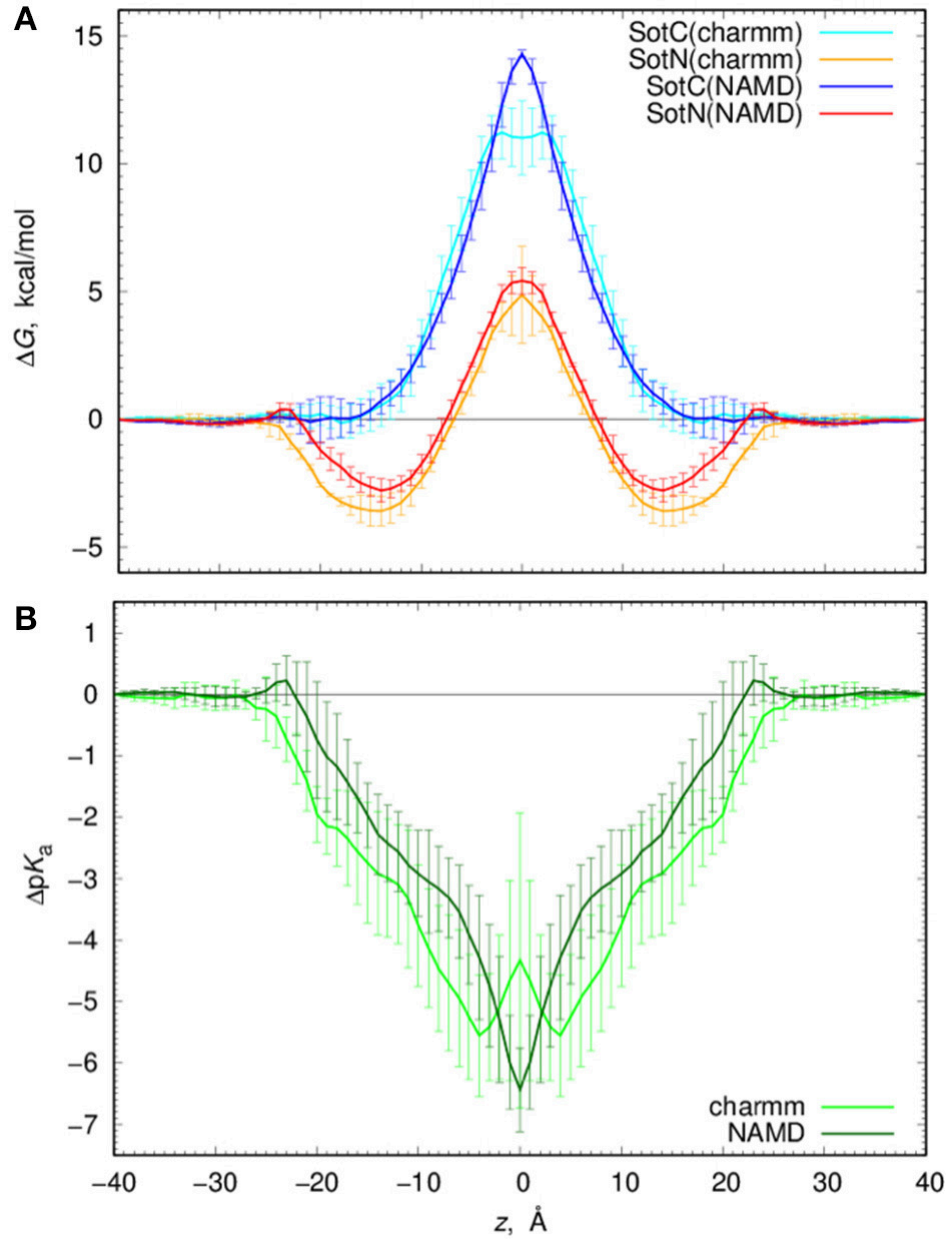
Analysis of d-sotalol thermodynamics from umbrella sampling MD simulations. **(A)** Free energy or potential of mean force (PMF) profiles for charged (SotC, blue and cyan) and neutral (SotN, red and orange) d-sotalol moving across a POPC membrane. In CHARMM simulations (cyan for SotC and orange for SotN) P2_1_ symmetry was used. See text for more details. **(B)** d-sotalol p*K*_a_ shifts computed from PMFs in **(A)**.

We computed free energy profiles for SotC and SotN moving across a POPC membranes based on analysis of drug position fluctuations around restrained *z* positions in US MD simulations as described above. Those profiles are shown in Figure 7A for both NAMD and CHARMM simulations. For SotN, differences between NAMD and CHARMM free energies are within uncertainties (shown as error bars in Figure 7A), obtained as measures of profile asymmetries (see Figure S9 and Supplemental text). However, for SotC the free energy barrier is ~3 kcal/mol smaller for CHARMM (11.2 ± 1.1 kcal/mol) compared to NAMD (14.4 ± 0.1 kcal/mol). Such free energy decrease along with a flat free energy profile for |*z*| < 3 Å can be due to P2_1_ point group transformations used in CHARMM simulations. This is also in line with interfacial connections to both bilayer interfaces seen in these simulations (see Figure 1 and discussion above). However, relatively large asymmetries of up to ~2 kcal/mol (Figure S9) preclude us from an unambiguous assignment of this difference.

If we compare SotC and SotN free energy profiles shown in Figure 7A, we will see differences such as substantially higher central peak for SotC, e.g., 14.4 vs. 5.4 kcal/mol for SotN from NAMD simulations, as well as presence of a deep interfacial minimum of −2.8 kcal/mol for SotN at |*z*| = 14 Å, similar to one seen for cationic cisapride (Figure 1 and discussion above). Such minimum indicates a substantial neutral drug accumulation at the water-membrane interface. Interestingly, there is practically no such minimum for SotC, although, a shallow ~-1 kcal/mol trough can be seen on a not-symmetrized PMF profile in Figure S9. The substantial difference in peak heights for SotC and SotN is not unexpected, however, and was also observed for basic amino acid side chains in our previous simulations (Li et al., 2008, 2013). It can be explained by different molecular mechanisms governing SotC and SotN permeation: substantial membrane deformations for the former and nearly complete drug dehydration for the latter (Vorobyov et al., 2010, 2014; Li et al., 2012, 2013). Based on free energy difference between charged and neutral drug forms we can also approximate p*K*_a_ shift and thus preferred protonation form of a drug across the membrane:

(9) $$\begin{array}{l} \Delta p{\text{K}}_{\text{a}}=1/\left(2.303{\text{k}}_{\text{B}}\text{T}\right)\;\left\{\Delta {\text{W}}_{\text{SotN}}\left(\text{z}\right)-\Delta {\text{W}}_{\text{SotC}}\left(\text{z}\right)\right\} \end{array}$$

where *k*_B_ is Boltzmann constant, *T*—absolute temperature and Δ*W* (*z*) are position-specific free energies for charged and neutral d-sotalol. Corresponding Δp*K*_a_ profiles are shown in Figure 7B and indicate rapid downward Δp*K*_a_ shifts soon after the drug gets into contact with membrane. Near the membrane center Δp*K*_a_ reaches about −6.5 for NAMD and −4.5 for CHARMM based calculations, with the latter estimate being smaller due to a ~3 kcal/mol smaller SotC free energy barrier discussed above. Qualitatively, both results are similar and indicate rapid drug deprotonation soon after a drug starts moving across a membrane. In fact, considering its first aqueous p*K*_a_ of 8.3, even getting as close as 20 Å to the membrane center will already lead to drug deprotonation. However, it should be noted that we have not considered a possible role of a zwitterionic d-sotalol form, SotZ, in this equilibrium.

### d-Sotalol water-membrane partitioning and permeations: connection to experiments

Next, we need to attempt connecting our findings to experimental observables such as water—membrane partitioning coefficient *K* and permeability rate *P*. All the relevant data are summarized in Table 1. There is an experimental estimate for water—dimyristoylphosphatidylcholine (DMPC) membrane *K*′(wat → mem) of 2.50 obtained at 303 K (Redman-Furey and Antinore, 1991). This is an apparent value, which takes into account a pH-dependent fraction of membrane-active drug species at those conditions. However, since we know that only SotN is expected to accumulate in the membrane we can compute an intrinsic *K*-value at experimental pH = 7.2 using drug aqueous p*K*_a_ = 8.37 and Henderson-Hasselbach equation to obtain *K*(wat → mem) = 2.50 ^*^ 10^(8.37−7.20)^ = 37.0. And corresponding partitioning free energy is Δ*G*(wat → mem) = −*RT* ln *K*(wat → mem) = −2.17 kcal/mol. These estimates, again, do not take into account a presence of SotZ form in the drug protonation equilibrium, which will likely further increase *K*-value and decrease corresponding Δ*G*. Nevertheless, we can compare experimental estimates with values we computed from NAMD US free energy profiles using Equations (3) and (4). Estimated *K*(wat → mem) and Δ*G*(wat → mem) values for SotN of 13.4 ± 8.6 and −1.6 ± 0.4 kcal/mol (see also Table 1), respectively, are in good agreement with experiment also considering a different lipid (POPC vs. DMPC) and temperature (310 vs. 303 K) used in simulations and experiment. Estimates from CHARMM simulations (Table S6) are similar, within an error of NAMD values. As expected, SotC does not accumulate in the membrane, with *K*(wat → mem) and Δ*G*(wat → mem) of 0.69 ± 0.36 and 0.23 ± 0.0.28 kcal/mol, respectively (Table 1).

**Table 1:** Water-membrane partitioning and permeability data from umbrella sampling MD simulations for charged (SotC) and neutral (SotN) d-sotalol translocation across a POPC membrane using NAMD.

	**Experiment**	**Umbrella sampling MD simulations**	
		**SotC**	**SotN**
*W*(peak), kcal/mol		14.38 ± 0.14	5.43 ± 0.53
|*z*(peak)|, Å		0.0	0.0
*W*(well), kcal/mol		−0.16 ± 0.10	−2.79 ± 0.47
|*z*(well)|, Å		32.5	14.0
*W*(barrier), kcal/mol		14.54 ± 0.17	8.22 ± 0.71
Δ*G*(wat->mem), kcal/mol	−2.17^a^	0.23 ± 0.28	−1.60 ± 0.37
*K*(wat->mem)	37^a^	0.69 ± 0.36	13.41 ± 8.58
*D*(wat), 10^−5^ cm^2^/s		0.99 ± 0.14	0.98 ± 0.06
*D*(mem), 10^−5^ cm^2^/s		0.061 ± 0.039	0.087 ± 0.020
log *P*(wat->mem), [log cm/s]	−6.50^b^	−8.57	−4.43

a *Redman-Furey and Antinore (1991) using pK_a_ = 8.3 to compute intrinsic values based on observed apparent K′(wat → mem) of 2.50*.

b *Liu et al. (2012) using measured PAMPA permeability rate*.

**Figure 8:**
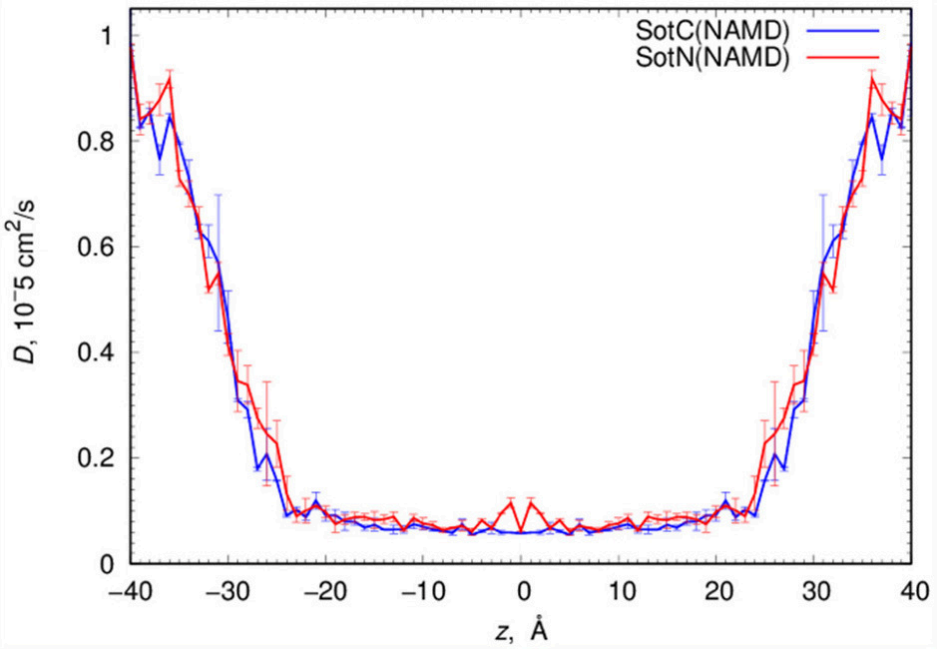
Analysis of d-sotalol diffusion from umbrella sampling MD simulations. Diffusion coefficient profiles are computed as described in the text. Error bars shown are computed from profile asymmetries.

MD simulations of water-membrane partitioning are a good test of the drug model accuracy, and can predict how much drug accumulates in the membrane compared to bulk water. However, it does not consider the kinetics of drug movement across a membrane, which is also essential for predicting its pharmacology and toxicology. Permeability rates, *P*, provide corresponding estimates and are measured experimentally using different cell lines such as caco-2 or artificial membrane systems such as PAMPA (Parallel Artificial Membrane Permeability Assay) (Bermejo et al., 2004). Experimental estimates for d-sotalol *P* are available from a recent study (Liu et al., 2012) with a PAMPA *P*-value of 3.2 × 10^−7^ cm/s. A direct comparison between experimental and computed *P* values is known to be challenging, with many complicating factors precluding direct quantitative assessment of absolute values (Carpenter et al., 2014; Di Meo et al., 2016; Bennion et al., 2017). Nevertheless, we computed *P* estimates for both SotC and SotN using Equation (8) as was done in our previous study (Vorobyov et al., 2014) based on free energy and diffusion coefficient profiles. The latter, shown in Figure 8, were obtained based on correlation times and mean fluctuations of drug COM in *z* direction using Equation (5) as was also done previously (Vorobyov et al., 2014). The computed diffusion coefficient profiles indicate a rapid 10-fold drop of diffusion coefficients for both SotC and SotN as drug molecules start interacting with lipid membranes, similar to many previous observations (Carpenter et al., 2014; Vorobyov et al., 2014). Interestingly, diffusion coefficients for SotC and SotN are similar, both in water and in the membrane interior (Figure 8 and Table 1), despite difference in net charge and very different drug—membrane interactions. Computed *P*-values, presented in Table 1 as log *P* of −8.57 for SotC, and −4.43 for SotN encompass an experimental estimate of −6.50. Based on those values alone, we cannot comment on accuracy of our prediction, and comparison with values for other drug molecules (desirably, with similar functionalities) as was done in a recent study (Bennion et al., 2017) would be the best. What our computed values indicate though, that a neutral drug is about 4-orders of magnitude more permeable compared to a cationic one, and that both values are within few orders of magnitude of an experimentally observed permeability.

### d-Sotalol—membrane interactions: effect of anionic lipids

Thus far, we only considered d-sotalol partitioning across a POPC membrane using US MD simulations for a single drug molecule. However, we also tested if lipid membrane composition affects drug—lipid interactions. In fact, cardiomyocyte lipid membrane is known to host multiple lipid types: in addition to dominant zwitterionic phosphatidylcholine and phosphatidylethanolamine, it also has a substantial fraction of anionic lipids—phosphatidylserine, phosphatidylinositol and phosphatidic acid [6–13% in human (Post et al., 1995) or 17–18% in feline cardiac cells (Leskova and Kryzhanovsky, 2011) based on total phospholipid content]. Anionic lipids are expected to increase membrane binding affinity for cationic drug forms and other cations, as was evidenced by our previous study where we saw increase in the interfacial binding for a positively charged arginine side chain analog, methyl guanidinium, in the presence of an anionic lipid phosphatidylglycerol (Vorobyov and Allen, 2011). The possible effect of anionic lipids on neutral drug binding is less clear and is worth testing as well. Therefore, we performed simulations of both SotC and SotN in lipid membranes containing 15% POPS and 85% POPC, respectively, and compared the results to corresponding drug simulations with pure POPC membranes.

**Figure 9:**
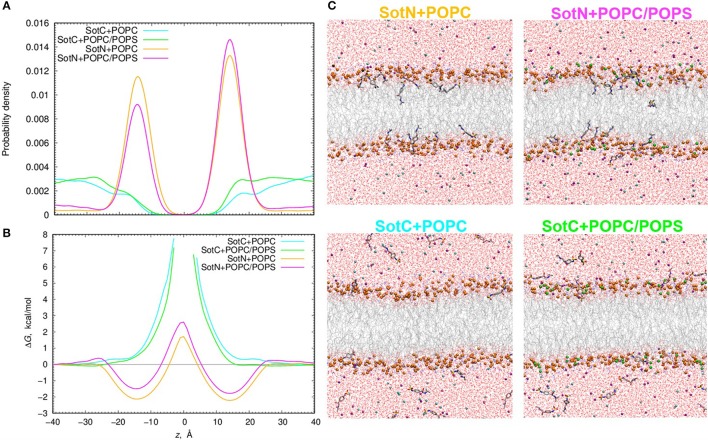
Analysis of d-sotalol partitioning in the presence of anionic lipids from unbiased MD simulations. **(A)** Probability density and **(B)** free energy or potential of mean force (PMF) profiles for charged (SotC) and neutral (SotN) d-sotalol moving across a 100% POPC lipid bilayer (cyan and yellow for SotC and SotN, respectively) or a bilayer composed of an 85% POPC and 15% POPS lipid mixture (green and magenta for SotC and SotN, respectively). **(C)** Molecular snapshots of equilibrated SotN+POPC, SotC+POPC, SotC+POPC/POPS systems after 500 ns, and SotN+POPC/POPS system after 1000 ns of unbiased MD simulations on the Anton 2 supercomputer. P atoms of POPC and POPS lipids are shown as orange and green balls, respectively. See Figure 1 caption for other molecular representation and coloring information.

We used 500 or 1000 ns long unbiased MD simulations with multiple (15–16) drug molecules initially placed in bulk aqueous solution, corresponding to ~40 mM drug concentration. Most SotN molecules become bound to the lipid membrane within 200 ns for the simulation with pure POPC and around 400 ns with a POPC/POPS mixture (see Figure S11). The equilibrium aqueous concentration of SotN drops to ~8 mM for POPC/POPS and ~5 mM for a POPC only system. For systems containing SotC, most drug molecules remain in aqueous solution throughout the simulations with only ~4 (out of 15) interacting with membrane regardless of the lipid composition (Figure S11). Equilibrated systems are shown in Figure 9C demonstrating substantial membrane binding of SotN but not of SotC. Drug probability distributions from those simulations, computed based on simulation data after equilibration (which was achieved in 200 or 400 ns), are shown in Figure 9A. These data confirm the picture demonstrating substantial interfacial binding for SotN with well-defined probability maxima around |*z*| = 15 Å for both POPC and POPC/POPS systems. No interfacial binding was detected for systems containing SotC (Figure 9A). In the cationic sotalol system with POPS present, there is a slightly increased accumulation of the drug density in |*z*| range of 15–30 Å compared to a system with POPC only. This can be due to expected attraction between anionic lipid head groups of POPS and positively charged SotC moieties. However, the effect is small and is thus unlikely to be physiologically significant in this case.

The probability distributions shown in Figure 9A can be converted to free energy profiles as Δ*G*(*z*) = –*k*_B_*T* ln ρ(*z*), where ρ is probability density, *k*_B_ is Boltzmann constant, and *T* is the absolute temperature (see also analogous Equation 4 above). Those profiles are shown in Figure 9B and are in general agreement with those from US MD simulations shown in Figure 7A previously. As expected, we did not observe SotC located near the membrane center during 500 ns of unbiased MD simulations, and therefore free energy profiles are not defined in this region. However, we observe that the slope of the profile is steeper in the presence of POPS, suggesting a higher translocation barrier and hence slower translocation in this case. SotN molecules were distributed throughout the membrane, and thus we could compute complete free energy profiles including central peaks. Interestingly, there are shallower interfacial binding troughs (by 0.5–0.6 kcal/mol at |*z*| = 14–15 Å), higher central peak (by ~1.1 kcal/mol) and thus larger translocation barriers in the presence of POPS, indicating less favorable membrane binding and slower translocation rates for SotN. Upon comparison of SotN free energy profiles from US and unbiased MD simulations, shown in Figure 7A, 9B, respectively, we observed a substantially smaller central free energy peak (by 3.7 kcal/mol) and shallower interfacial binding (by 0.6 kcal/mol) in unbiased simulations. There are several factors which can contribute to such differences, including multiple drug molecules, larger membrane patch, and presence of applied electric field in unbiased MD simulations, all of which can possibly lead to smaller permeation barriers. A detailed elucidation of these and other factors is beyond the scope of this study and will be investigated in our subsequent works.

## Discussion

### Exploring ionized drug membrane partitioning

At physiological pH many cardiac channel blockers exist in aqueous solution mostly in their cationic form for d-sotalol and cisapride, and zwitterionic form for moxifloxacin (ionized, but with net zero charge). Our MD simulations have demonstrated that all of them cause substantial membrane deformations, with lipid head groups and water molecules coordinating them deep into the hydrophobic membrane core. Large free energy barriers occur at the center of the membrane as a result of the deformations, making such mode of drug translocation unlikely. Moreover, ionized d-sotalol and moxifloxacin do not demonstrate any interfacial membrane binding, indicating that they will not be accumulating there, and thus limiting their protein target accessibility through this route. Interfacial membrane binding is, however, possible for cationic cisapride, and its accumulation there could play a role in its pharmacological profile. However, to provide a more complete picture for drug membrane translocation and membrane-mediated protein target affinity, additional less-polar drug protonation states should be considered. This is what we did for d-sotalol; a prominent example of high-arrhythmia risk hERG blocker. At a physiological pH of 7.4, 89% of this drug exists in a cationic form, indicating a ~1.3 kcal/mol energetic penalty for its deprotonation, which can be easily overcome by the hydrophobic environment of lipid membranes that provide a barrier for charged and polar species (Gennis, 1989).

### Computing charged and neutral d-sotalol membrane partitioning

In addition to a cationic d-sotalol force field model, we developed parameters for one of the neutral forms of d-sotalol. SotN is substantially more lipophilic, as expected, with a free energy penalty near the membrane center of ~5 kcal/mol, compared to ~15 kcal/mol barrier for the cationic species, which, interestingly, correlates with the ratio of their dipole moments. Moreover, unlike SotC, SotN accumulates at the water-membrane interface, making it accessible for binding to protein targets through the lipophilic pathway. Such accumulation, which can be quantified by water-membrane partitioning coefficient, *K*(wat → mem), is in agreement with experiment (within an uncertainty, see Table 1), suggesting a good quality of the developed empirical model.

Also, SotN does not lead to substantial membrane perturbations; it transiently coordinates with only a few water molecules as it moves across a hydrophobic core of a membrane, unlike SotC. This entails different molecular mechanisms of membrane translocation: a traditional “solubility-diffusion” for SotN governed by drug dehydration, and so called “ion induced defect” for a cationic form, where a cost of membrane deformation plays a major role as was suggested in our previous studies on charged amino acid side chain and small hydrophilic ion translocation (Li et al., 2012; Vorobyov et al., 2014). Thus, we can expect very different dependence of their membrane translocation energetics on lipid membrane composition, such as a strong decrease with a corresponding reduction in membrane thickness for SotC, but not for SotN. This is why we expect good agreement with experiment for SotN water-membrane partitioning despite using a different lipid bilayer (POPC vs. DMPC). Translocation of SotC, however, is expected to be very sensitive to the mechanical properties of membrane such as thickness, as well as the presence of cholesterol, or polyunsaturated fatty acid tails, which can increase or reduce membrane rigidity, respectively (Feller et al., 2002; Pitman et al., 2004). Our computed membrane translocation energetics for charged cisapride and neutral d-sotalol across POPC membrane are very similar, but we expect a larger barrier for cisapride in thicker and/or cholesterol-containing membranes. This will lead to different modulation of drug accessibility for intracellular and membrane-located protein targets. As a first step toward the investigation of lipid composition dependence, we briefly examined the role of anionic lipids in water-membrane d-sotalol partitioning energetics. Despite expected more favorable drug membrane binding in the presence of POPS, we observed an opposite trend with shallower interfacial troughs for SotN and larger translocation barriers for both SotN and SotC. This indicates that such modulation can be due to specific drug—membrane interactions rather than a general electrostatic attraction.

### Estimating d-sotalol membrane permeation kinetics

For SotC and SotN, we also provide estimates of membrane translocation kinetics, expressed as permeability rates (*P*). SotN has a translocation rate that is four orders of magnitude faster than SotC (see Table 1), which is as expected from the difference in their membrane translocation energetics, and similarly reduced diffusion coefficients in the membrane interior. This is in agreement with our previous estimations for ions (Vorobyov et al., 2014) and other drugs (Boiteux et al., 2014). Based on the computed *P*-values and the membrane thickness considered in those calculations, we estimate that a single SotN molecule can translocate between interfacial binding sites on opposite sides of the membranes in about 7.5 × 10^−3^ s (millisecond time range), whereas for SotC crossing membrane will take around 150 s. SotN is expected to be accumulated in the membrane over 10-fold compared to its equilibrium concentration in bulk aqueous solution, which is why we are considering its permeation, even though it is a minor component in the bulk aqueous solution at the physiological pH, regardless of its unknown ratio to a membrane-impermeable zwitterionic form. An experimental *P* estimate for d-sotalol based on measurements using PAMPA is in between our computed values for SotC and SotN (see Table 1 and Liu et al., 2012). Yet, a direct numerical comparison of our computed and experimental *P* estimates is extremely challenging, as has been indicated in many previous studies (Orsi et al., 2009; Carpenter et al., 2014; Di Meo et al., 2016; Bennion et al., 2017). This is largely because experimentally measured quantities mostly represent so-called apparent values, which typically include contributions from different drug protonation forms at experimental pH, depend on water layer thickness and condition, and may encompass different drug permeation routes (Bermejo et al., 2004; Avdeef et al., 2005; Ottaviani et al., 2006; Orsi et al., 2009). More standardized intrinsic *P*-values for neutral drug forms are typically harder to get (Bermejo et al., 2004; Orsi et al., 2009), and even then, quantitative agreement with MD computed values remains challenging due to substantial differences between an experimental macroscopic system, and a microscopic molecular model. Therefore, an agreement between relative *P*-values for different drugs is typically sought (Orsi et al., 2009; Carpenter et al., 2014; Bennion et al., 2017), which will be explored in our future studies.

### Predicting possible membrane-mediated ion channel accessibility pathways

One mode of ion channel block by drugs is through an intracellular aqueous pathway, where a drug in the cytosol passes through a channel lower gate, when it is open, and occludes a channel pore (Hille, 2001). Another possible mechanism for ion channel block is through a lipophilic route, which was observed in a recent MD study for a local anesthetic, benzocaine, entering a central pore of sodium voltage-gated channel Na_v_Ab via lipid-facing channel openings (fenestrations) (Boiteux et al., 2014). In the case of the hERG blocker d-sotalol studied here, SotN would likely to be a dominant drug form binding to the channel via this route, but it could become protonated again once it is in the pore.

Our recent combined experimental/computational studies of pH- and state-dependent hERG block by another high-risk pro-arrhythmic drug dofetilide (sharing the same functional groups as d-sotalol, but more potent) suggested that drug protonation equilibrium plays a crucial role in its channel binding affinity (Wang et al., 2016). To the best of our knowledge, no such studies have been done for d-sotalol yet. The experimentally measured on-rate of d-sotalol binding to hERG is quite slow, in the range of several minutes (Numaguchi et al., 2000). This is consistent with our computed membrane permeation rate for cationic d-sotalol form. Recent experimental studies using cells pre-equilibrated with sotalol, i.e., after the drug crossing cell membranes, demonstrate faster than 200 ms hERG block (Li et al., 2017; Windley et al., 2017), indicating that drug membrane permeation could be a rate-limiting step considering preferential drug channel access via the intracellular aqueous pore. However, other reasons for such outcome, such as a preponderance of a lipophilic channel access pathway from of a local membrane bound pool of the drug, suggested by our neutral d-sotalol simulations, are possible and can be tested by additional experiments as well as comprehensive drug—channel MD simulations. This along with pH-dependent measurements can help elucidating roles of different drug protonation states and their contribution to channel block.

Moreover, experimental drug—channel on-rates, which are crucial components of functional scale kinetic models used for *in silico* evaluation of pro-arrhythmia proclivities (as in the CiPA initiative), can be corroborated using atomistic MD simulations, such as those presented in this study. Moreover, atomistic MD simulations can be used to identify different drug—channel interaction pathways not easily discernable via experiment alone. For instance, through comparison of computed rates for drug membrane translocation and binding to the channel via aqueous and lipophilic pathways, we can predict likely rate limiting step, and relative contributions of all those processes to experimentally measured rates, thus informing kinetic models and likely improving their accuracy and predictive power. The spatially resolved ionization-state-specific drug localization profiles and water—membrane permeation rates computed here represent the first crucial step toward this goal.

Further insight into structural determinants of drug-induced channel blockade, including possible drug access pathways, can be provided by comprehensive mutagenesis studies, similar to one done recently for a large set of congenital long QT syndrome 2 associated hERG mutations (Anderson et al., 2014). Though not directly related to drug-induced hERG block, several mutations that were implicated in directly affecting channel gating or permeation were for hERG residues facing the water-membrane interface (Lees-Miller et al., 2015; Saxena et al., 2016), and therefore would be easily accessible by drugs like neutral d-sotalol, and cationic cisapride, that we explored in this study.

Even more importantly, similar computational approaches can be used as one of the steps to design drugs, which have similar membrane binding affinities and bind around mutated protein residues that result in altered channel function. Such an approach focusing on a desired drug lipophilicity and spatial arrangement of crucial functional groups was used, for instance, to design selective sodium channel blockers (Muraglia et al., 2007; De Luca et al., 2012; De Bellis et al., 2017). Such a structure-function based approach was shown to improve drug safety profile through mitigation of off-target effects, including hERG block (De Bellis et al., 2017).

### Limitations and future directions

Our study represents just a first step in atomistic-level elucidation of thermodynamics and kinetics of cardiac channel blocking drug translocation across a lipid membrane. We obtained reasonable free energy profiles and water-membrane partitioning coefficients with a moderate amount of sampling (10–15 ns per umbrella sampling window or 0.8–1.2 μs for entire simulations). In some cases, we had to run additional simulations with alternative initial drug orientations to compensate for their slow reorientation in the membrane interior observed in our study. More extensive simulations for other drug membrane partitioning using a different empirical force field and molecular modeling software were reported recently (Bennion et al., 2017). They can potentially provide improved accuracy provided a high quality of an underlying empirical model and sufficient sampling of drug tumbling, and thus can be considered as a viable alternative of our approach. In our future studies we will also test several alternative options for enhanced sampling (Bernardi et al., 2015) such as metadynamics (Barducci et al., 2011), which has been recently used in membrane partitioning simulations to properly sample degrees of freedom orthogonal to the reaction coordinate and thus provide a more accurate energetics (Jambeck and Lyubartsev, 2013).

Alternatively, replica exchange simulations can be employed, which can be especially useful for modeling mixed membrane systems (Huang and Garcia, 2014). In our study, we mostly used a one component lipid membrane containing POPC, whereas lipid composition of cellular membranes is much more complex. For instance, plasma membranes of cardiomyocytes (where hERG channels are mostly located) has substantial fractions of zwitterionic posphatidylcholine, phosphatidyethanolamine, and sphingomyelin, negatively charged phosphatidylserine and non-polar cholesterol with substantial differences in their distribution between inner and outer leaflets (Post et al., 1995). This is without taking into account lateral membrane heterogeneity and existence of functional microdomains such as lipid rafts and caveolae, suggested to influence cardiac ion channel function (Maguy et al., 2006). At this time, however, we are not yet in position to study such complex heterogeneous systems via atomistic simulations, but coarse-grained models, such as a popular MARTINI force field (Marrink and Tieleman, 2013), are well-suited for such investigations and can be potentially used for studying cardiac drug interactions with realistic lipid membranes. In terms of atomistic simulations, we are planning to extend our studies to simulate drug partitioning to binary mixtures of phospholipids and cholesterol, which is expected to substantially influence ionizable drug partitioning and permeation, as discussed above. Another direction, which we already started exploring here, are binary mixtures of two phospholipids with different head groups, possibly influencing drug permeation kinetics and thermodynamics via specific interactions and/or altering membrane physical-chemical properties.

Estimated drug permeation rates and their relation to experimentally measured quantities remain uncertain as was mentioned above. In this study we could only compare relative values for cationic and neutral d-sotalol, which encompass an experimental estimate. However, it is not clear if we can simply relate those values to a measured apparent permeability via computing effective resistances to permeation as was done in a recent study (Carpenter et al., 2014). Another pertinent issue is computing permeability rates for drug molecules with pronounced interfacial binding (such as neutral d-sotalol in this study), which will clearly increase a barrier height a drug will need to hop over to permeate as was noted previously (Orsi et al., 2009). Therefore, an expression for permeability rates (Marrink and Berendsen, 1994) traditionally used for their calculations for polar and ionic species, where free energies are referenced to bulk aqueous solution, might not work anymore. In this study for SotN we used a variant of this expression with free energy set to 0 at the interfacial binding site and computing permeability just across a central barrier. A validity of such approximation remains to be seen in more thorough investigations, e.g., by comparing results with drug translocation rates computed from long unbiased MD simulations. Moreover, this approach does not take into account drug translocation between the interfacial binding site and bulk water. This contribution becomes dominant for hydrophobic drugs such as general anesthetics (Vorobyov et al., 2012), not considered in this study.

For d-sotalol and other hERG blockers with sulfonamide functional group (e.g., dofetilide, ibutilide, E4031), an unresolved issue is its anionic, deprotonated drug fraction, such as one in the zwitterionic d-sotalol (SotZ). A neutral sulfonamide group has been thoroughly parameterized recently and is included in the generalized CHARMM force field (Yu et al., 2012), whereas no atom type for anionic N or any associated parameters are available to the best of our knowledge. For d-sotalol in water at the physiological pH, a cationic form with a neutral sulfonamide group is a dominant form, with SotZ and/or SotN having a ~11% contribution. Based on our prediction, only SotN can move across a membrane, but we need to know SotZ and SotN ratios in order to relate computed membrane partitioning energetics to experimental observables. Moreover, SotZ can be potentially an important contributor to hERG binding through the interactions of its negatively charged sulfonamide functionality with basic residues in the voltage-sensing domain (VSD) of a channel, for example. Such interactions were revealed in a recent crystallographic/electrophysiological study in a VSD of a voltage gated Na_v_1.7/Na_v_Ab chimera channel, where an anionic sulfonamide “warhead” directly and selectively interacts with a gating charge carrying arginine residue, immobilizing a voltage sensor in its activated state (Ahuja et al., 2015). Whether a similar binding motif is possible for hERG remains to be seen, but it should not be discounted, and thus accurate empirical force field for an anionic sulfonamide functionality will need to be developed and can be validated on predicting an aforementioned drug—channel interaction.

Sotalol is a chiral molecule, and in this work we only studied one enantiomer: d-sotalol, which was used in an infamous SWORD clinical study (Waldo et al., 1996) mentioned above. Sotalol enantiomers can be synthesized and separated (Carr et al., 1991; Foster and Carr, 1992; Brodfuehrer et al., 1997), however, a racemic mixture of d- and l-isomers has been used in many biophysical, physiological and pharmacological experimental studies up to date. l-sotalol is known to have some beta-blocking activities, whereas d-sotalol seems to be inert (Gomoll and Bartek, 1986) (a reason why it was used for SWORD study), but they share very similar electrophysiological properties, including QT prolongation (Touboul, 1993; Manoach and Tribulova, 2001). Even though interaction between two chiral molecules, e.g., sotalol and lipid, can be different for stereoisomers (and used for their separation), we do not expect substantial changes for l-sotalol—membrane interactions as they are mostly governed by dehydration for a neutral drug or membrane deformation by a charged drug electric field. Therefore, simulations with d-sotalol should be sufficient, however, a more complex situation will arise for drug—channel interaction simulations, where both stereoisomers might need to be tested.

Nevertheless, despite the limitations of this study, related to tested drug and membrane models, our work demonstrated good agreement between computed and experimental data, and can definitely be used to predict the molecular mechanisms, energetics and kinetics of drug-membrane interactions, and potentially ion channel binding pathways. Moreover, the presented study can be used, for instance, for informing multi-scale kinetic models of cardiovascular (and other) drug effects on cellular, tissue and organ levels (Clancy et al., 2016), as was done in our recent study, where we modeled charged and neutral flecainide (cardiac sodium channel blocker with some pro-arrhythmic proclivity) effects (Yang et al., 2016). We are planning a similar extension of the current study along with atomistic structure based investigations of sotalol interactions with hERG using a combination of molecular docking and all-atom molecular dynamics simulations. Several other drugs with different hERG affinities and pro-arrhythmia proclivities will be investigated as well for both lipid membrane and hERG binding assays.

## Author contributions

IV, CC, and SN: designed the research; KD, SB, and IV: performed the research and analyzed data; All authors prepared the manuscript and approved the final submitted version.

### Conflict of interest statement

The authors declare that the research was conducted in the absence of any commercial or financial relationships that could be construed as a potential conflict of interest.

## Abbreviations

aLQTS: acquired Long QT syndrome
CGENFF: CHARMM generalized force field
CHARMM: Chemistry at Harvard Molecular Mechanics
CiPA: comprehensive *in vitro* pro-arrhythmic assay
CisC: cationic cisapride
COM: center of mass
Cryo-EM: cryo-electron microscopy
DMPC: dimyristoylphosphatidylcholine
ECG: electro-cardiogram
GPU: Graphics Processing Unit
hERG: human *Ether-à-go-go*-Related Gene
K_v_: voltage gated potassium channel
LQTS: Long QT syndrome
MD: molecular dynamics
MM: molecular mechanics
MoxZ: zwitterionic moxifloxacin
PBC: periodic boundary conditions
PMF: potential of mean force
POPC: 1-palmitoyl-2-oleoyl-phosphatidylcholine
POPS: 1-palmitoyl-2-oleoyl-phosphatidylserine
QM: quantum mechanics
SotA: anionic d-sotalol
SotC: cationic d-sotalol
SotN: neutral d-sotalol
SotZ: zwitterionic d-sotalol
US: umbrella sampling
VSD: voltage sensing domain
WHAM: weighted histogram analysis method.
