# Supplementary material for: Digging into Lipid Membrane Permeation for Cardiac Ion Channel Blocker d-Sotalol with All-Atom Simulations
Source: Front Pharmacol. 2018 Feb 1;9:26. doi: 10.3389/fphar.2018.00026 (PMC5799612; doi:10.3389/fphar.2018.00026)
Supplement: Supplementary file 1 [file Presentation1.pdf]

Supplementary Information for

Digging into Lipid Membrane Permeation for Cardiac Ion Channel Blocker d-Sotalol with All-atom Simulations

Kevin R. DeMarco[1,2,3], Slava Bekker[1,4], Colleen E. Clancy[1,2], Sergei Y. Noskov[5] and Igor Vorobyov[1,2*]

[1]Department of Physiology and Membrane Biology, University of California at Davis, Davis, California, United States of America.

[2]Department of Pharmacology, University of California at Davis, Davis, California, United States of America.

[3]Biophysics Graduate Group, University of California at Davis, Davis, California, United States of America.

[4]Hartnell College, Salinas, California, United States of America.

[5]Centre for Molecular Simulations, Department of Biological Sciences, Faculty of Science, University of Calgary, Calgary, Alberta, Canada.

[*] Correspondence to: Igor Vorobyov, Ph.D.

Department of Physiology and Membrane Biology

University of California, Davis

4303 Tupper Hall, One Shields Ave.

Davis, CA 95616-8636

Email: ivorobyov@ucdavis.edu

Phone: +1-530-752-3932

Supplementary Text

Sotalol solvation and protonation energetics based on implicit solvent QM calculations

We used quantum mechanical (QM) calculations in vacuum as well as in widely used implicit solvent model SMD (Marenich et al., 2009) with results shown in Supporting Tables S1 and S2. According to MP2/6-31G(d) QM calculations in vacuum SotN form is favored over SotZ by around 47 kcal/mol. This preference drops to ~30 kcal/mol in hexadecane, a membrane-mimetic alkane and to just ~1.3 kcal/mol in water due to substantially more favorable solvation free energy for SotZ. However, even in water the neutral species is slightly favored over the charged one (**Table S1**). We also tested the robustness of such assessment using HF, MP2 and a popular hybrid density functional method B3LYP levels of theory and two basis sets: standard 6-31G(d) and substantially more complex aug-cc-pVDZ, as shown in Supporting Table 2. In most cases, SotN is still predicted to be favored in water with the only exception of B3LYP/aug-cc-pVDZ calculations predicting nearly equal stabilities of SotZ and SotN (**Table S2**). These calculations do not guarantee accurate assessment since they do not take into account specific solute-solvent interactions explicitly, but nevertheless provide us with a valuable approximation in the absence of experimental data.

Comparison of aqueous and hexadecane solvation free energies obtained via QM calculations helped us predict energetic penalties for different sotalol ionization forms as they transfer from water to a non-polar hydrophobic environment such as that of membrane interior (**Table S1**). All the ionized drug species, i.e. SotC, SotZ and SotA, are expected to have very large water-hydrocarbon translocation penalties, exceeding 30 kcal/mol, whereas that for SotN is predicted to be ~8 kcal/mol. Interestingly, SotZ is predicted to have a larger energetic penalty than either SotA or SotC despite the fact that the latter two forms carry a net charge. The high energetic cost for SotZ is commensurate with its huge dipole moment, which is almost twice the magnitude of the dipoles for cationic or anionic forms. All dipole moments were computed with the same drug orientation and position for all of them.

Accuracy of $P2_1$ point group (symmetry) application for CHARMM MD simulations of drug - lipid membrane systems

Two US simulations for each drug form (SotC and SotN) were performed – using NAMD and CHARMM programs with the same empirical force field models for all the system components and practically the same simulation parameters. A major difference is that in CHARMM simulations we used $P2_1$ symmetry, which allows sampling of lipid redistribution between bilayer leaflets (Dolan et al., 2002) to maintain the same cross-sectional area of the membrane as the drug moves across it. In the absence of $P2_1$ symmetry an area difference may arise between the top and bottom leaflets over the course of drug translocation. A strain contribution that stems from the area difference can increase free energy barrier, and $P2_1$ symmetry might help alleviate this problem. Indeed, we observed some variation in the average

number of lipids in the top and bottom leaflets, which is small when a drug is in bulk water and can reach up to 4 lipids, when it is in the membrane interior (see top panels in **Figure S3**). We also tested if P2_1 symmetry can lead to additional membrane distortions and thus measured average area per lipid and membrane thickness (middle and bottom panels in **Figure S3**), both of which are in good agreement with experimental values at 303 K, 64.3±1.3 Å^2 and 39.1±0.78 Å, respectively (Kucerka et al., 2011). Deuterium order parameter profiles, $|S_{CD}|$, shown in **Figure S4**, are also in good agreement with experimental estimates (Ferreira et al., 2013).

Drug reorientation sampling

We found that hindered drug tumbling discussed in the main text can lead to substantial asymmetries in the PMF profiles, especially for SotC. In our original NAMD and CHARMM US MD simulations we obtained asymmetries as high as 4 and 12 kcal/mol (**Figure S9**), which are clearly unacceptable for any simulation analysis. We found that such asymmetries are mostly caused by SotC being stuck in a particular orientation with respect to membrane normal and perturbing the membrane by forming an interfacial connection with water and lipid head groups. Since the cationic ammonium group is a main attractor of polar moieties in SotC, the interfacial connection forms with the side of the membrane the functional group is pointing toward. Due to a random initial orientation of drug molecules and their slow reorientation in the membrane interior, drug molecules may form interfacial connections with an opposite bilayer leaflet. For instance, a drug with COM at z = 2 Å can coordinate water molecules and lipid head groups from the lower leaflet. Such interfacial connection will likely lead to high strain energy due to substantial membrane perturbations required. Thus, we need to balance this out with connections to an adjacent bilayer leaflet for the same drug position. This can be done in several ways as was described in our previous study on arginine side chain membrane partitioning (Li et al., 2008). Here for CHARMM simulations, we created additional US windows for $|z| \leq 3$ Å by starting with the neighboring windows with interfacial connection to an opposite bilayer leaflet. This led to a reduction in asymmetry from ~12 to ~3 kcal/mol (**Figure S9**). For SotC NAMD simulations a different approach was used where a second set of US windows for $|z| \leq 20$ Å (roughly corresponding to POPC membrane thickness) was generated with a different initial drug orientation, namely rotated around z-axis by 180°. Using two sets of simulations for central US windows also helped to substantially reduce asymmetry from ~4 kcal/mol to nearly 0 kcal/mol in this case (see **Figure S9**). Interestingly, such approach worked differently for SotN NAMD simulation leading to an increase in asymmetry in bulk solvent by ~1 kcal/mol but a similar decrease in the interfacial binding region (**Figure S9**). Free energy profiles shown in **Figure 7A** in the main text included this additional sampling except for SotN CHARMM run, where such simulations were not performed. We also tested slightly different approach, were we flipped initial drug orientation around the xy plane by 180° for central windows ($-20 \leq z \leq 20$ Å for SotC and $-4 \leq z \leq 4$ Å for SotN). PMF convergence plots with those simulations included are shown in **Figure S10**. There is ~2 kcal/mol asymmetry for

SotC and practically no asymmetry for SotN simulations. Final symmetrized profiles from those runs (from 4 to 15 ns) are nearly identical to ones shown in main text **Figure 7A**.

Supplementary References

Dolan, E.A., Venable, R.M., Pastor, R.W., and Brooks, B.R. (2002). Simulations of Membranes and Other Interfacial Systems Using P21 and Pc Periodic Boundary Conditions. *Biophysical Journal* 82, 2317-2325.

Ferreira, T.M., Coreta-Gomes, F., Ollila, O.H., Moreno, M.J., Vaz, W.L., and Topgaard, D. (2013). Cholesterol and POPC segmental order parameters in lipid membranes: solid state 1H-13C NMR and MD simulation studies. *Phys Chem Chem Phys* 15, 1976-1989.

Kucerka, N., Nieh, M.P., and Katsaras, J. (2011). Fluid phase lipid areas and bilayer thicknesses of commonly used phosphatidylcholines as a function of temperature. *Biochim Biophys Acta* 1808, 2761-2771.

Li, L., Vorobyov, I., and Allen, T.W. (2008). Potential of mean force and pKa profile calculation for a lipid membrane-exposed arginine side chain. *J Phys Chem B* 112, 9574-9587.

Marenich, A.V., Cramer, C.J., and Truhlar, D.G. (2009). Universal solvation model based on solute electron density and on a continuum model of the solvent defined by the bulk dielectric constant and atomic surface tensions. *J Phys Chem B* 113, 6378-6396.

Redman-Furey, N.L., and Antinore, M.J. (1991). Determination of partition coefficients between dimyristoylphosphatidylcholine and water using differential scanning calorimetry. *Analytica chimica acta* 251, 79-81.

Supplementary Tables

Table S1. Relative QM energies and dipole moments for different d-sotalol protonation states from MP/6-31G(d) calculations in vacuum and implicit solvent using SMD model.

	sotC	sotZ	sotN	sotA	sotZ - sotN
**vacuum**					
ΔE, kcal/mol					47.15
μ, Debye	15.50	28.59	5.63	16.86	
**hexadecane**					
ΔE, kcal/mol					30.27
ΔG_{solv}, kcal/mol	-36.58	-27.04	-10.17	-30.65	-16.88
μ, Debye	17.15	32.67	6.16	18.47	
**water**					
ΔE, kcal/mol					1.30
ΔG_{solv}, kcal/mol	-70.52	-63.87	-18.02	-66.23	-45.85
μ, Debye	20.98	39.78	7.88	22.04	

Table S2. Relative QM energies for charged (SotC) and zwitterionic (SotZ) d-sotalol protonation states in vacuum and implicit water using PCM and SMD models. All energies are in kcal/mol. Gas-phase values are shown in red.

QM level of theory		***Relative energies and free energies (kcal/mol)***		
**geometry optimization**	**single-point energy**	**Δ*E*(SotZ-SotN)**	**ΔG_{solv}(SotZ)**	**ΔG_{solv}(SotN)**
HF/6-31G(d)	HF/6-31G(d)	51.79		
HF/6-31G(d)	HF/6-31G(d) PCM	13.43	-50.79	-12.43
HF/6-31G(d)	HF/6-31G(d) SMD	5.33	-65.29	-18.83
HF/6-31G(d) SMD	HF/6-31G(d) SMD	1.67	-71.34	-21.22
MP2/6-31G(d)	MP2/6-31G(d)	47.15		
MP2/6-31G(d)	MP2/6-31G(d) SMD	5.59	-57.89	-16.33
MP2/6-31G(d) SMD	MP2/6-31G(d) SMD	1.30	-63.87	-18.02
MP2/6-31G(d)	MP2/aug-cc-pVDZ	44.29		
MP2/6-31G(d)	MP2/aug-cc-pVDZ SMD	3.33	-56.46	-15.49
B3LYP/aug-cc-pVDZ	B3LYP/aug-cc-pVDZ	42.91		
B3LYP/aug-cc-pVDZ	B3LYP/aug-cc-pVDZ SMD	4.54	-55.33	-16.96
B3LYP/aug-cc-pVDZ SMD	B3LYP/aug-cc-pVDZ SMD	-0.23	-61.71	-18.57

Table S3. Partial atomic charges for charged (SotC) and neutral (SotN) d-sotalol models. (Optimized charge values are shown by asterisk)

Drug	Total Charge	Drug	Total Charge
**SotC**	1	**SotN**	0
S	0.426	S	0.426
O1	-0.381	H1	0.090
O2	-0.381	O1	-0.381
N1	-0.376	O2	-0.381
N2	-0.456	N1 *	-0.071
C1 *	-0.155	N2	-0.456
C2 *	0.295	C1 *	0.113
C3	0.267	C2 *	-0.261
C4 *	0.190	C3 *	-0.102
C5	-0.110	C4	0.006
C6	-0.110	C5	-0.110
C7	-0.265	C6	-0.110
C8	-0.265	C7	-0.279
C9	0.220	C8	-0.279
C10	-0.116	C9	0.220
C11	-0.116	C10	-0.116
C12	-0.017	C11	-0.116
O3	-0.650	C12	-0.017
H1	0.320	H2	0.258
H2	0.320	H3	0.317
H3	0.317	O3 *	-0.704
H4	0.090	H4	0.090
H5	0.090	H5	0.090
H6	0.090	H6	0.090
H7	0.090	H7	0.115
H8	0.115	H8	0.115
H9	0.115	H9	0.090
H10	0.090	H10	0.090
H11	0.090	H11	0.090
H12	0.090	H12	0.090
H13	0.090	H13	0.090
H14	0.090	H14	0.090
H15	0.090	H15	0.115
H16	0.115	H16	0.115
H17	0.115	H17	0.090
H18	0.090	H18	0.090
H19	0.090	H19	0.090
H20	0.090	H20	0.413
H21	0.413		

Table S4. Gas-phase cationic d-sotalol (SotC) – water interactions.

	Water interaction energy (kcal/mol)			Water interaction distance (Å)		
	IE(QM)	IE(MM)	|ΔIE|	*R*(QM)	*R*(MM)	|Δ*R*|
N2	-1.72	-1.92	0.20	3.29	3.29	0.00
O1	-9.28	-11.17	1.89	3.09	3.19	0.10
O1	-5.82	-6.57	0.75	2.97	3.02	0.05
O1	-7.40	-8.72	1.32	3.06	3.16	0.10
O2	-6.48	-7.36	0.87	3.05	3.15	0.10
O2	-4.91	-5.81	0.89	3.02	3.07	0.05
O2	-3.96	-4.88	0.92	3.10	3.25	0.15
H1	-17.22	-16.14	1.09	1.92	1.97	0.05
H2	-6.24	-4.93	1.31	3.05	3.40	0.35
H3	-10.69	-8.10	2.58	1.97	2.02	0.05
H4	-8.62	-7.11	1.51	2.38	2.78	0.40
H5	-9.81	-9.48	0.34	2.23	2.63	0.40
H6	-8.88	-8.24	0.63	2.63	2.93	0.30
H7	-9.56	-8.83	0.74	2.28	2.68	0.40
H10	-7.34	-5.87	1.47	2.36	2.71	0.35
H11	-8.72	-7.82	0.90	2.45	2.80	0.35
H12	-7.11	-5.26	1.85	2.34	2.74	0.40
H13	-7.45	-6.02	1.43	2.38	2.73	0.35
H14	-7.12	-5.21	1.91	2.34	2.74	0.40
H15	-8.09	-6.69	1.39	2.37	2.72	0.35
H18	-5.26	-2.75	2.51	2.27	2.67	0.40
H19	-5.36	-3.31	2.05	2.30	2.70	0.40
H20	-5.66	-3.11	2.55	2.40	2.80	0.40
H21	-13.70	-10.76	2.94	1.84	1.99	0.15
**RMSE**			**1.59**			**0.29**

Table S5. Gas-phase neutral d-sotalol (SotN) – water interactions.

	**Water interaction energy (kcal/mol)**			**Water interaction distance (Å)**		
	IE(QM)	IE(MM)	|ΔIE|	*R*(QM)	*R*(MM)	|Δ*R*|
N1	-0.86	-1.03	0.18	5.04	5.29	0.25
N2	-4.46	-3.08	1.38	3.20	3.30	0.10
O1	-5.84	-5.89	0.04	3.04	3.14	0.10
O2	-5.59	-5.49	0.10	3.04	3.14	0.10
O3	-6.23	-7.73	1.50	3.03	3.03	0.00
H1	-1.898	-2.035	0.137	2.673	2.873	0.2
H2	-4.71	-6.41	1.71	2.27	2.27	0.00
H3	-6.61	-5.76	0.86	1.98	2.03	0.05
H4	-2.10	-2.02	0.09	2.70	2.90	0.20
H5	-1.73	-0.73	1.00	2.98	3.33	0.35
H6	-1.59	-1.43	0.16	2.81	2.96	0.15
H9	-1.33	-1.21	0.13	2.79	2.94	0.15
H10	-2.82	-1.37	1.45	2.78	2.93	0.15
H11	-1.44	-0.78	0.65	2.75	3.00	0.25
H12	-1.44	-1.64	0.20	2.74	2.89	0.15
H13	-1.58	-0.98	0.61	2.71	2.96	0.25
H14	-2.07	-2.15	0.08	2.70	2.85	0.15
H17	-2.92	-1.64	1.28	2.34	2.74	0.40
H18	-2.66	-2.00	0.66	2.38	2.78	0.40
H19	-4.60	-4.60	0.00	4.42	4.57	0.15
**RMSE**			**0.83**			**0.21**

Table S6. Water-membrane partitioning data from CHARMM umbrella sampling MD simulations for charged (SotC) and neutral (SotN) d-sotalol translocation across a POPC membrane

	experiment	SotC			SotN		
W(peak), kcal/mol		11.23	±	1.05	4.90	±	1.92
$\|z$(peak)$\|$, Å		2.0			0.00		
W(well), kcal/mol		-0.15	±	0.59	-3.61	±	0.56
$\|z$(well)$\|$, Å		18.00			14.00		
W(barrier), kcal/mol		11.39	±	1.20	8.51	±	2.00
ΔG(wat->mem), kcal/mol	-2.17 [a]	0.31	±	0.21	-2.50	±	0.43
K(wat->mem)	37 [a]	0.60	±	0.23	58.14	±	46.46

[a] from ref. (Redman-Furey and Antinore, 1991) using pK_a =8.3 to compute intrinsic values based on observed apparent K'(wat→mem) of 2.50.

Supplementary Figures

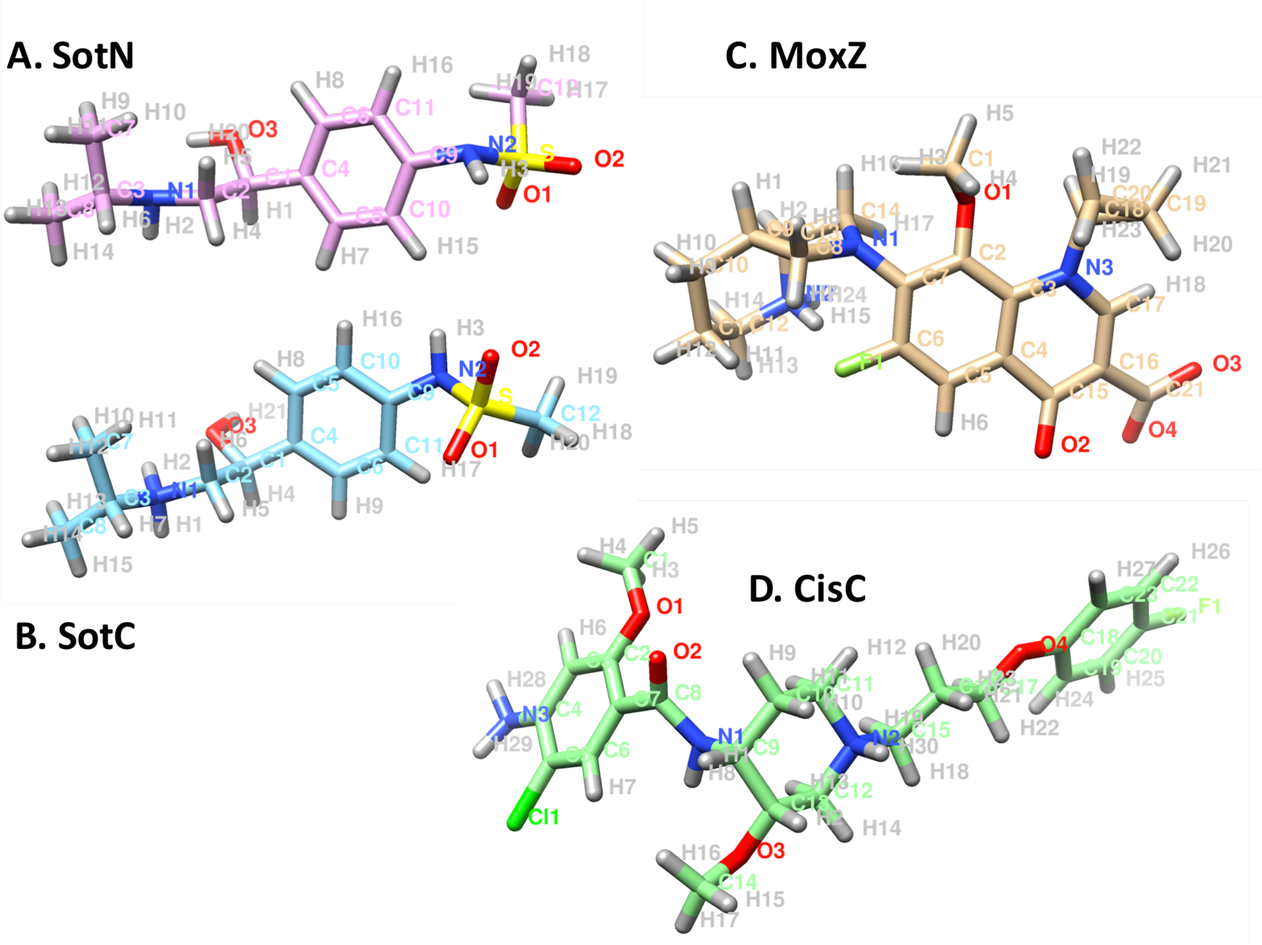

Figure S1. Atom naming for different drug molecules. SotC – cationic d-sotalol, SotN – neutral d-sotalol, CisC – cationic cisapride, MoxZ – zwitterionic moxifloxacin.

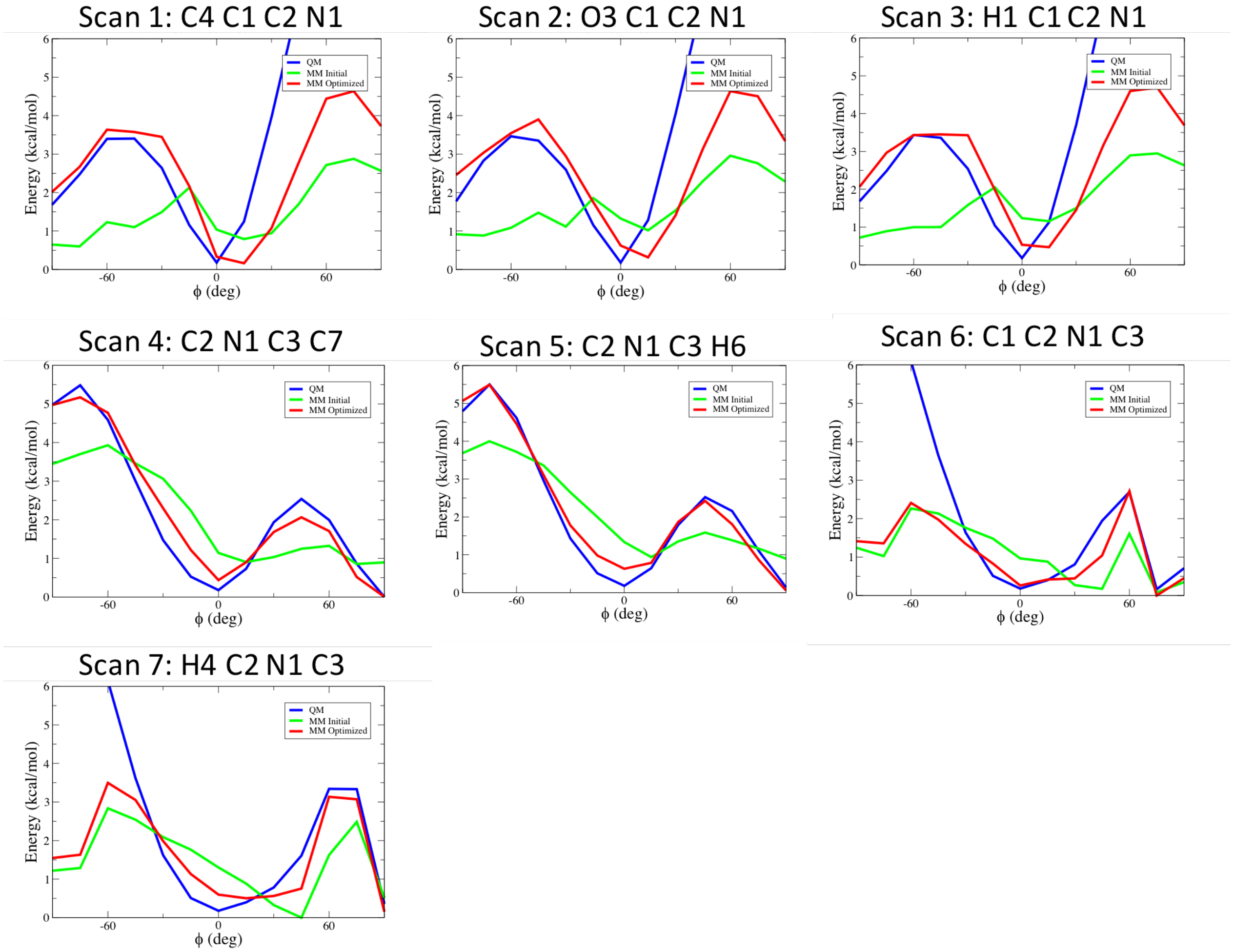

Figure S2. Gas-phase torsional energy profiles for neutral sotalol (SotN) from quantum mechanical (QM), initial and optimized molecular mechanics (MM) calculations. Atom names correspond to ones in Figure S1A as well as topology and parameter files in the Appendix.

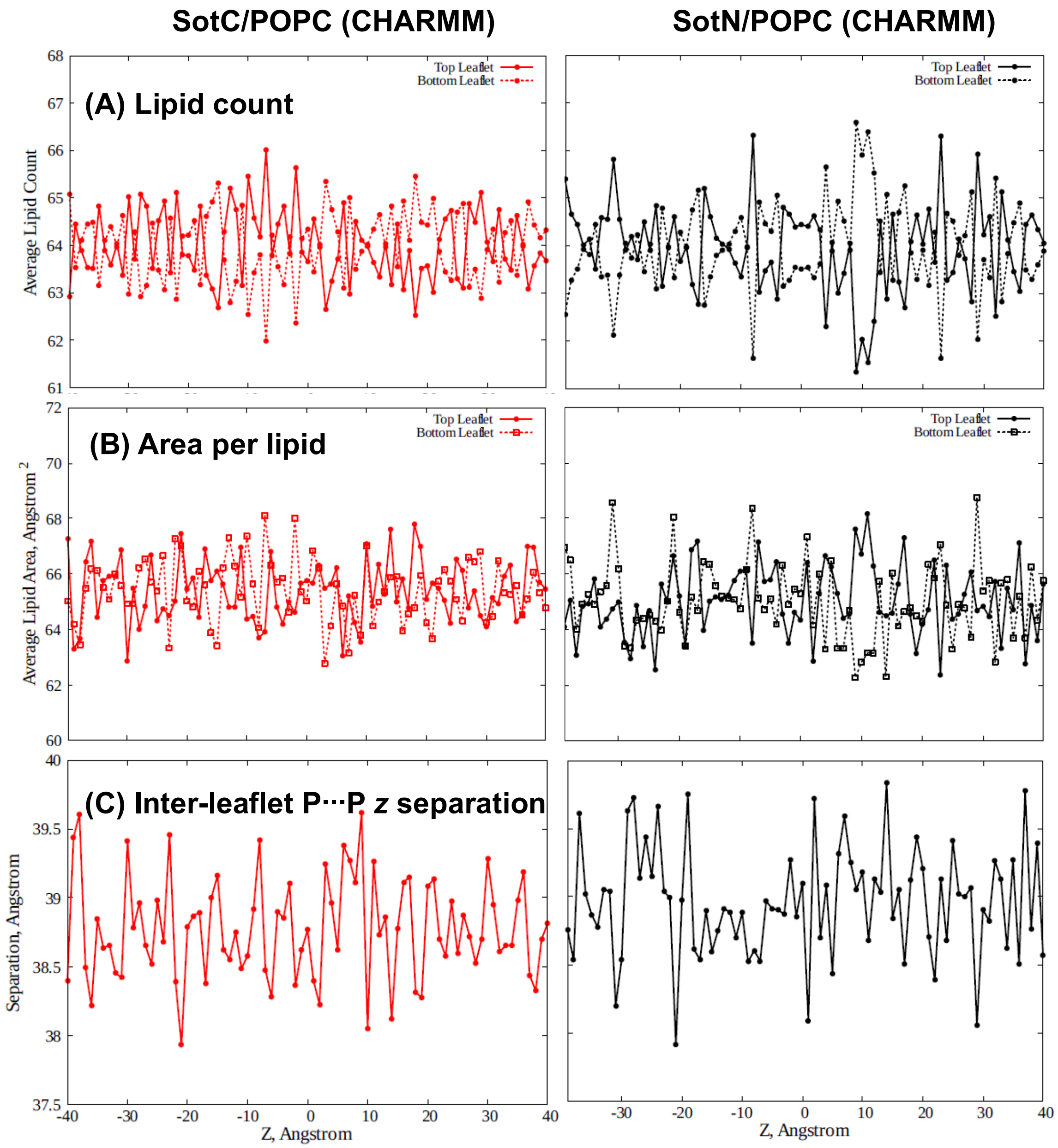

Figure S3. POPC lipid bilayer properties from d-sotalol partitioning CHARMM umbrella sampling MD simulations using P2$_1$ symmetry as a function of drug *z* position across the membrane. (A) Average numbers of lipids in top and bottom leaflets of the bilayer. (B) Average areas per lipid in top and bottom leaflets of the bilayer. (C) Average *z* distances between P lipid atoms in two leaflets, representing membrane thicknesses.

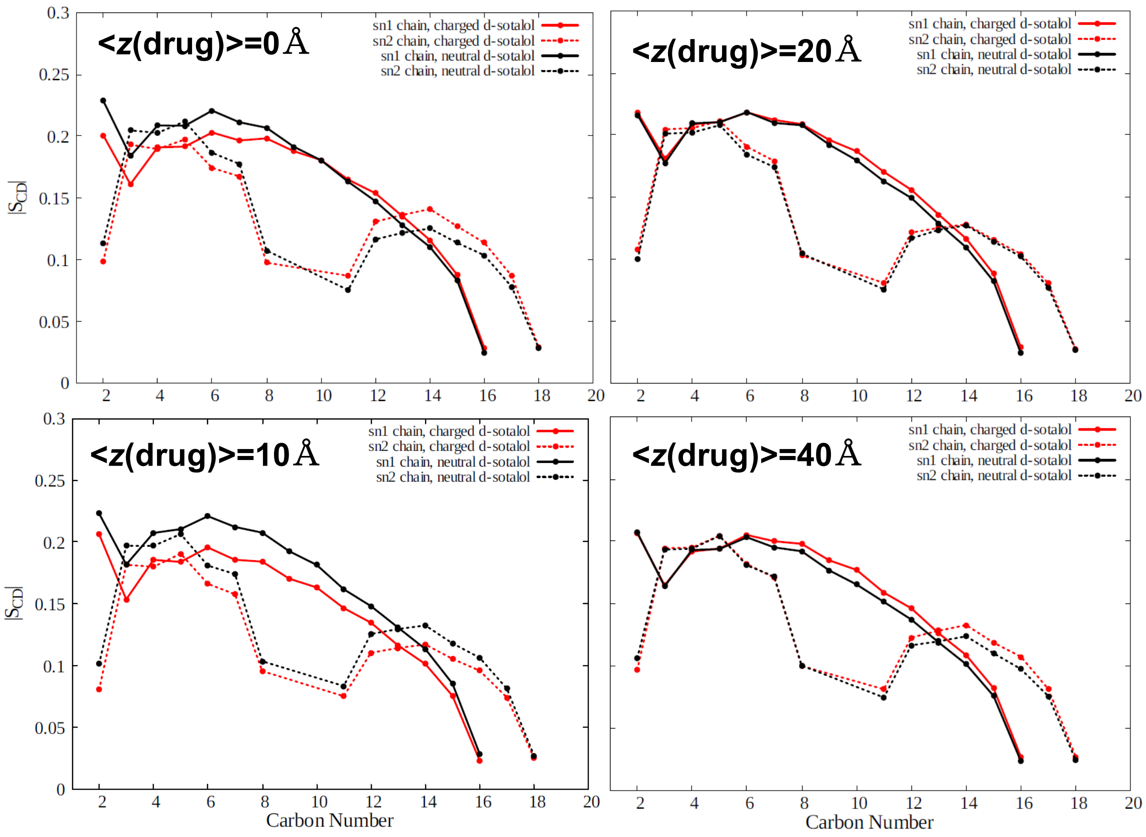

Figure S4. POPC lipid tail order parameter profiles from d-sotalol partitioning CHARMM umbrella sampling MD simulations using P2$_1$ symmetry for several drug *z* positions across the membrane.

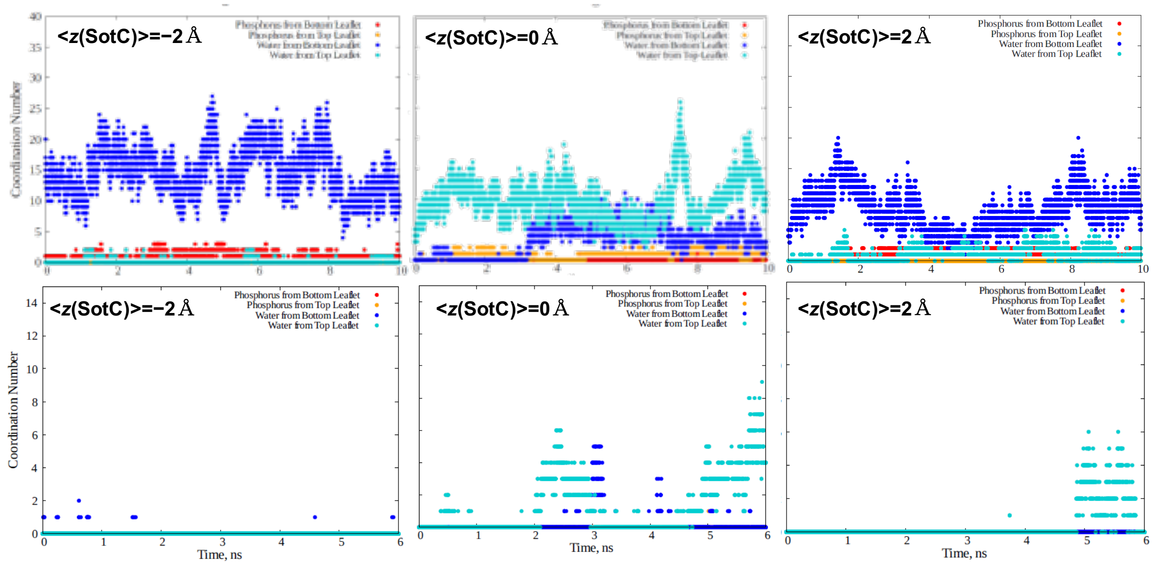

Figure S5 d-sotalol solvation numbers from hydrated membrane system components (water and lipid P atoms) as a function of simulation time from CHARMM umbrella sampling MD simulations. Solvation numbers were computed as number of water O or lipid P atoms within 5.0 Å of d-sotalol non-hydrogen atoms.

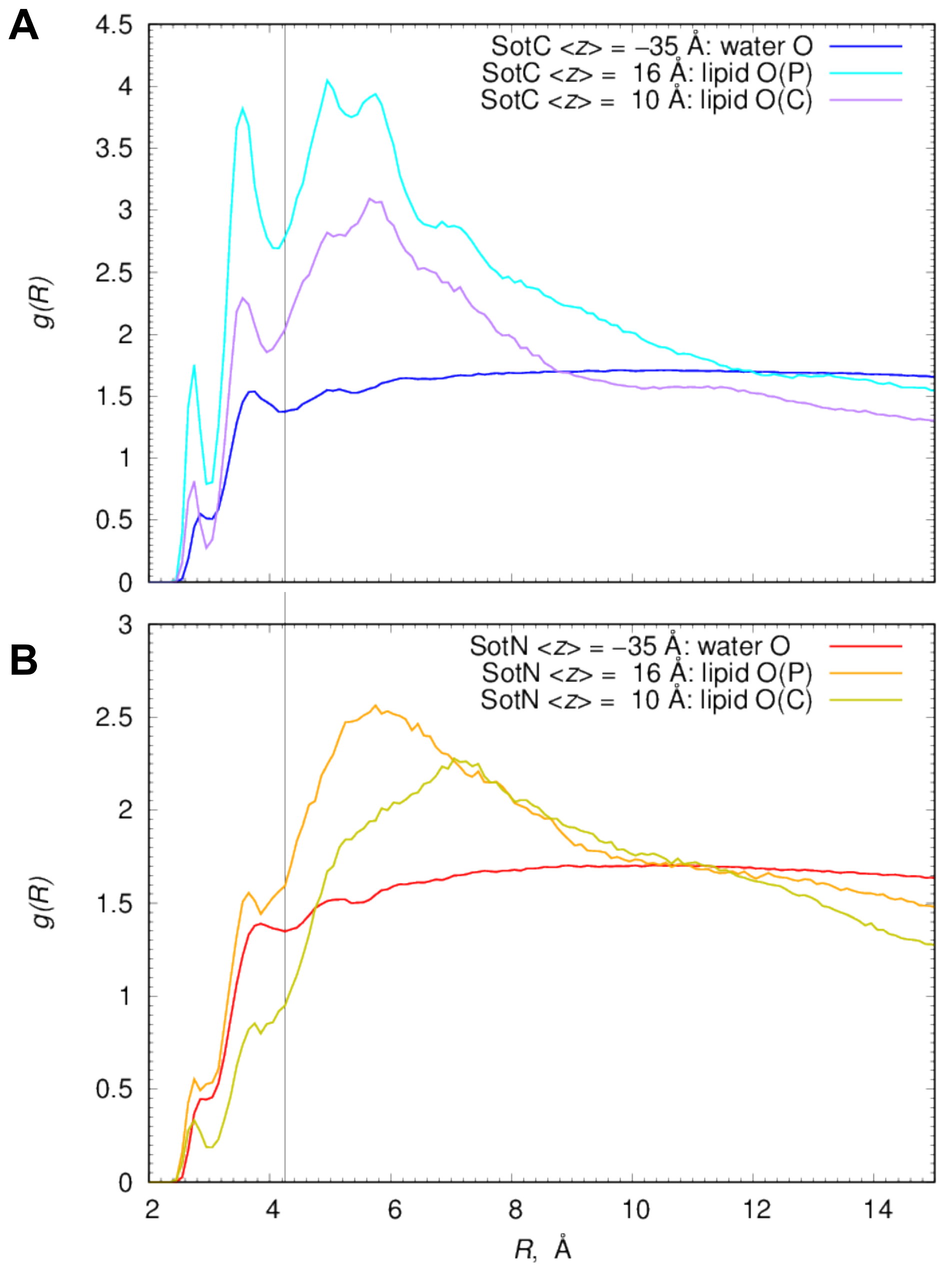

Figure S6. Several representative radial distribution function (RDF) g(*R*) profiles for d-sotalol non-hydrogen atom interactions with O atoms of water, lipid phosphate (P) and ester (C) functionalities from umbrella sampling simulations of drug POPC membrane translocation. A black vertical line indicates a cutoff distance of 4.25 Å used to compute solvation numbers in Figure 5 of the main text. It mostly coincides with RDF minimum for water O and shifted by just 0.1-0.2 Å from the corresponding RDF minima for lipid phosphate and ester oxygens.

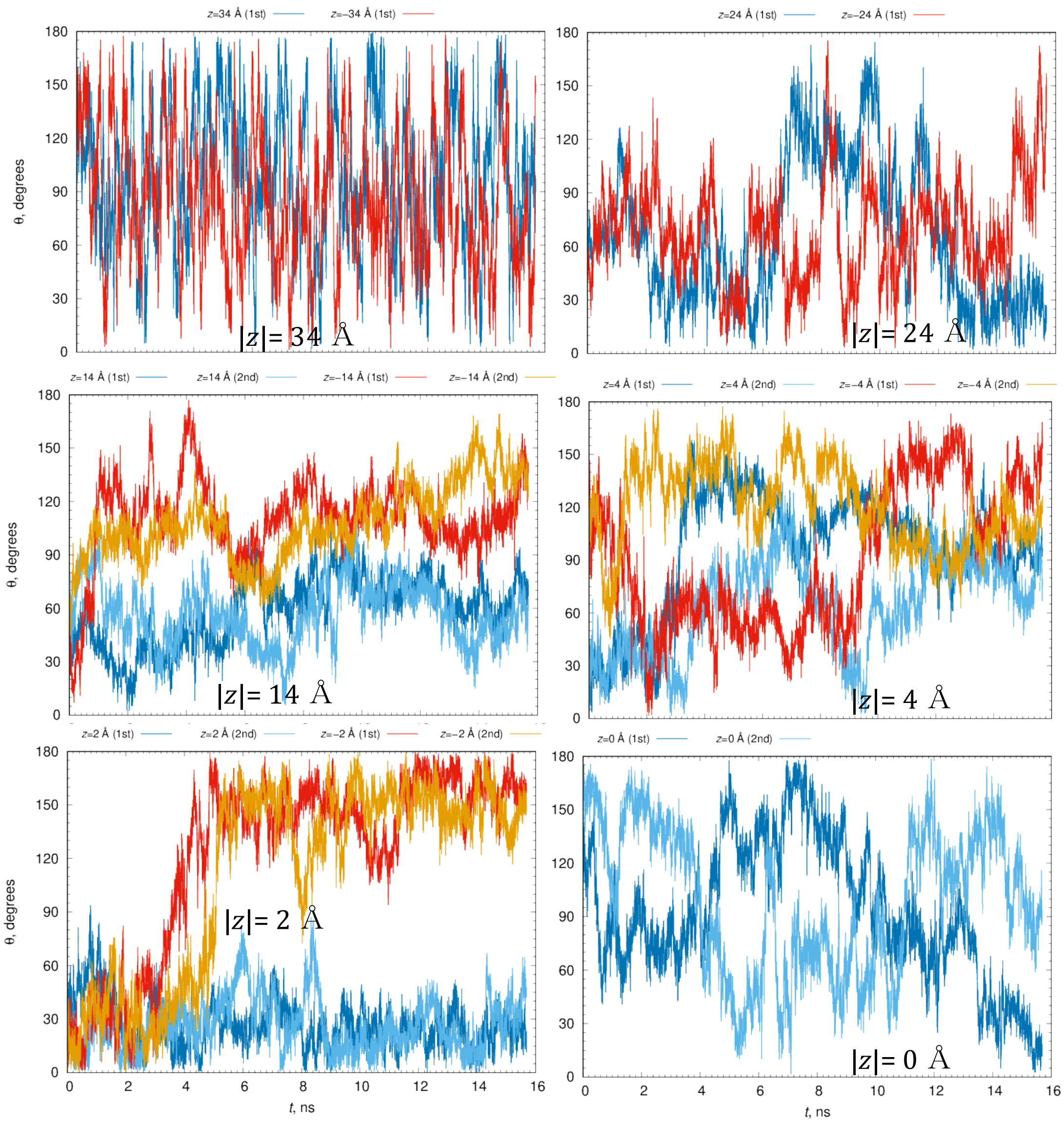

Figure S7. Time series for the polar angle θ representing orientation of neutral d-sotalol (SotN) N1···S vector with respect to membrane normal for selected windows from umbrella sampling MD simulations using NAMD. Profiles from both original runs (1st) and ones started from a drug re-oriented around the *z* axis (2nd) are included.

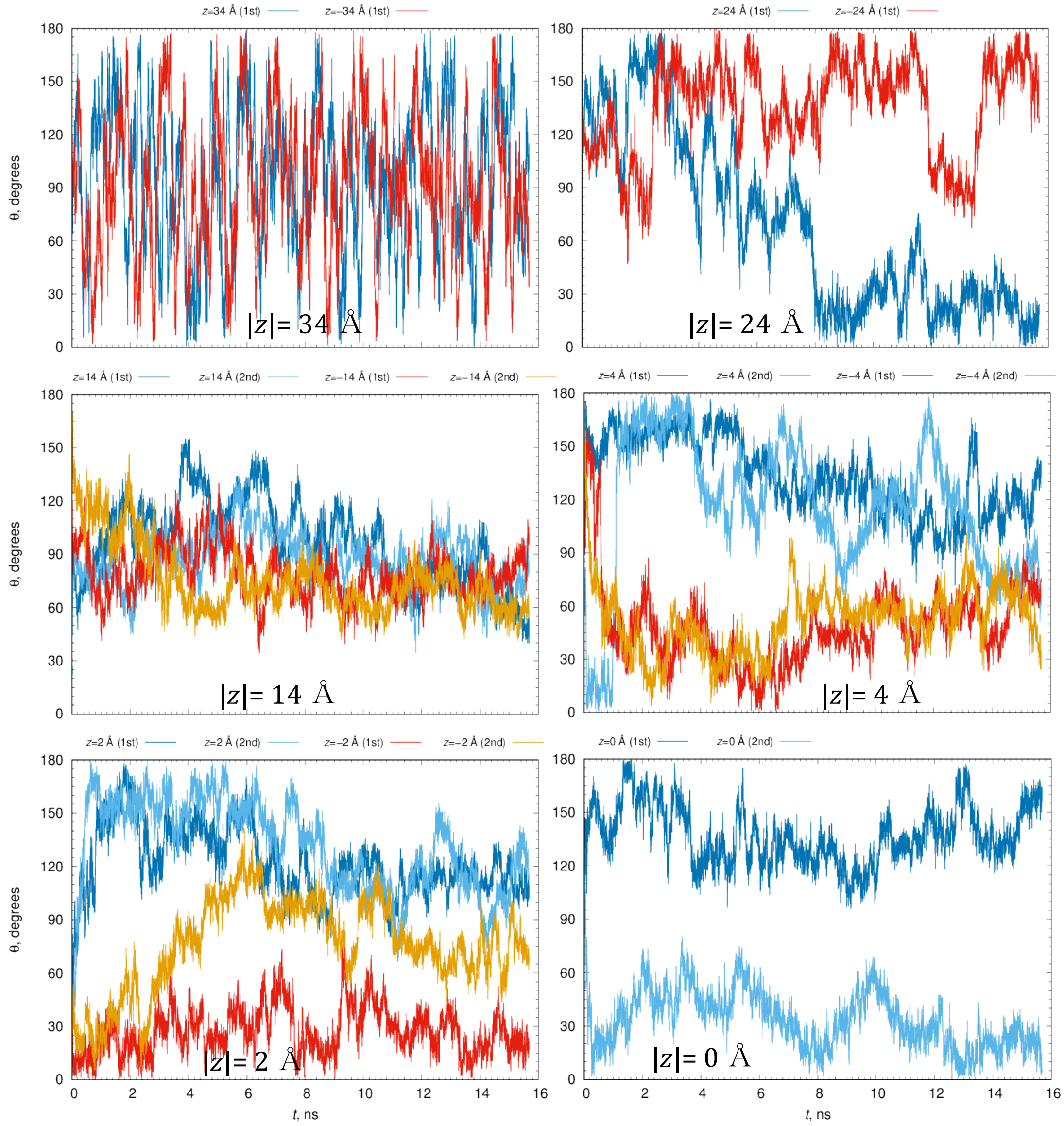

Figure S8. Time series for the for the polar angle θ representing orientation of cationic d-sotalol (SotC) N1···S vector with respect to membrane normal for selected windows from umbrella sampling MD simulations using NAMD. Profiles from both original runs (1st) and ones started from a drug re-oriented around the *z* axis (2nd) are included.

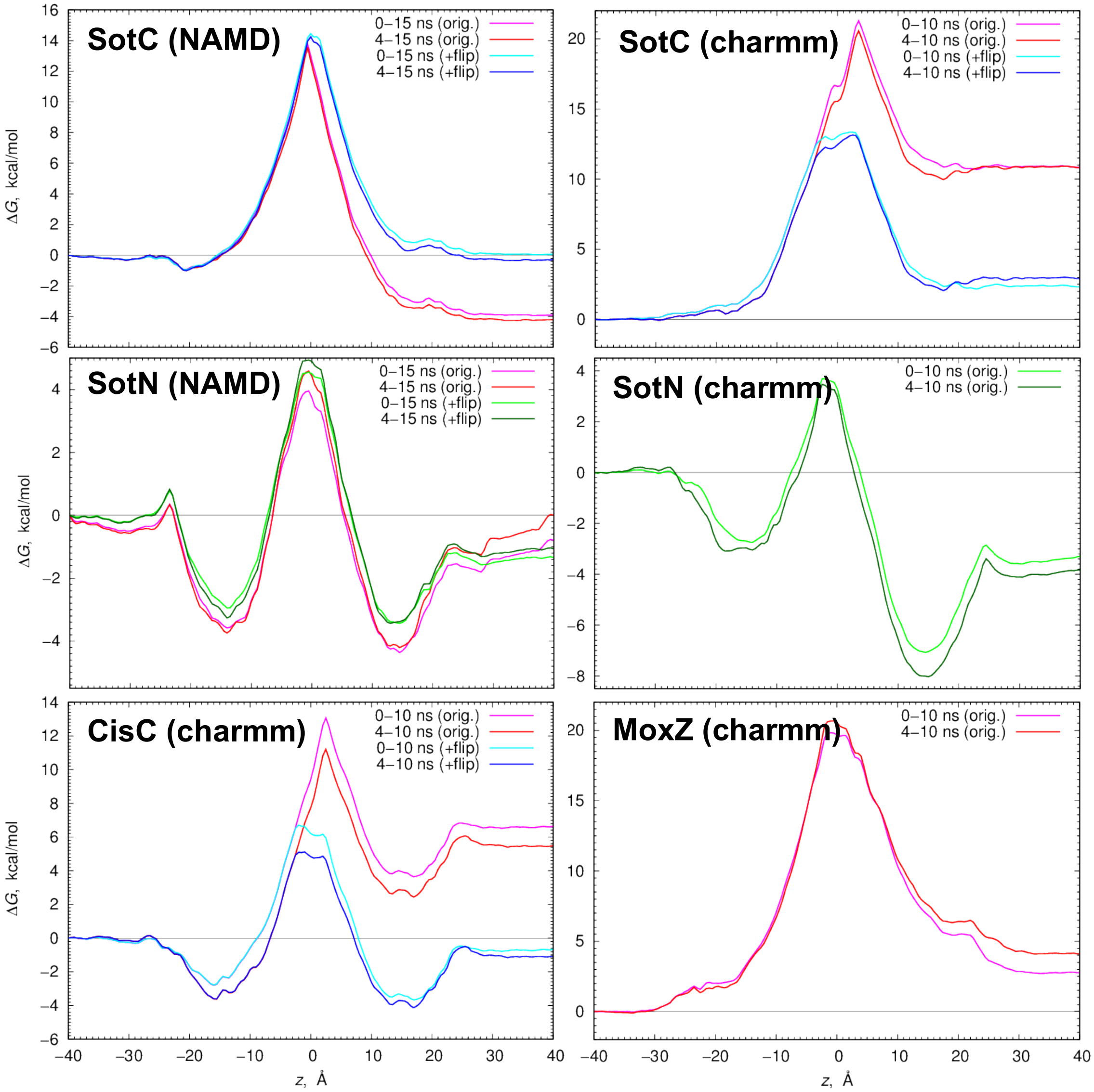

Figure S9. Free energy or potential of mean force (PMF) profiles for drug crossing a POPC membrane computed from umbrella sampling MD simulations. Non-symmetrized profiles are shown. They were computed using weighted histogram analysis method (WHAM) either using all the simulation data or discarding first 4 ns for each umbrella sampling window based on solvation number profiles in Figure S5. In some cases, additional simulations with initial drug orientation rotated around the *z* axis (indicated as +flip) were performed to improve drug re-orientation sampling.

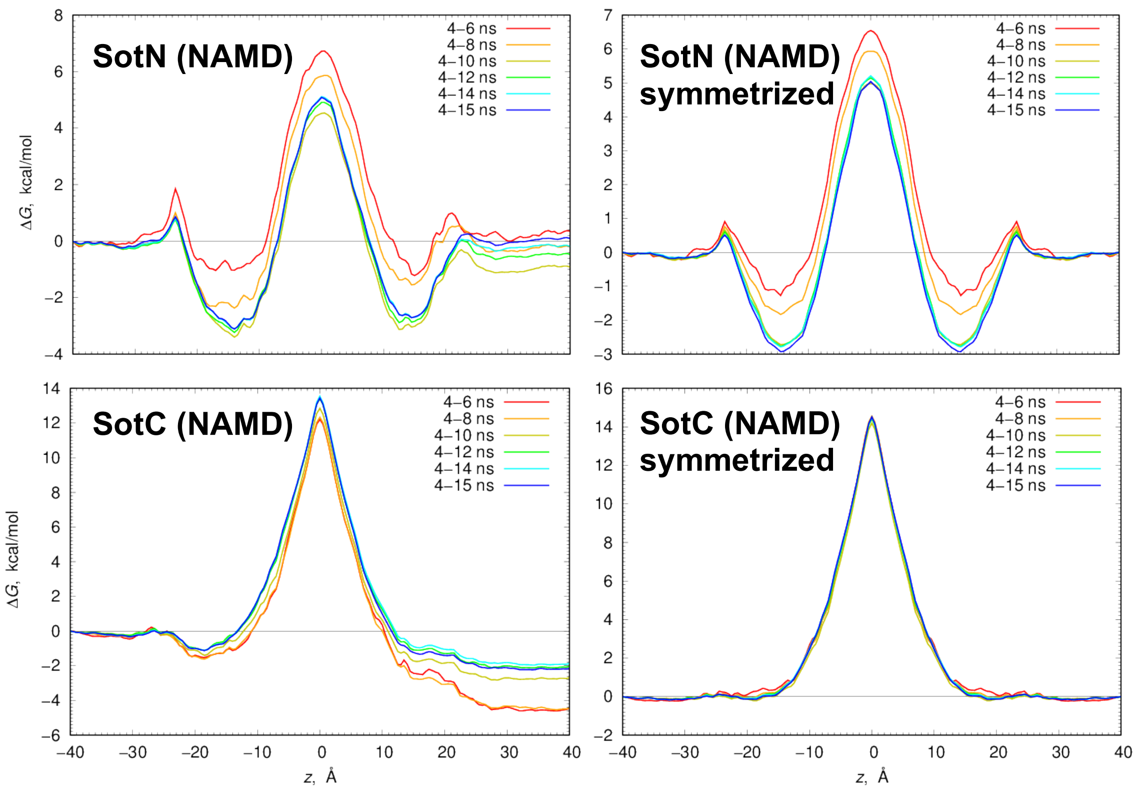

Figure S10. Convergence of free energy or potential of mean force (PMF) profiles for cationic (SotC) or neutral (SotN) d-sotalol crossing a POPC membrane computed from umbrella sampling MD simulations using NAMD. Both non-symmetrized (left) and symmetrized (right) profiles are shown. They were computed using weighted histogram analysis method (WHAM) discarding first 4 ns for each umbrella sampling window based on solvation number profiles in Figure S5. Additional simulations with initial drug orientation flipped around the *xy* plane were included for central windows (-20≤z≤20 Å for SotC and -4≤z≤4 Å for SotN) were performed to test drug re-orientation sampling. Final symmetrized profiles from those runs (from 4 to 15 ns) are nearly identical to ones shown in main text Figure 7A.

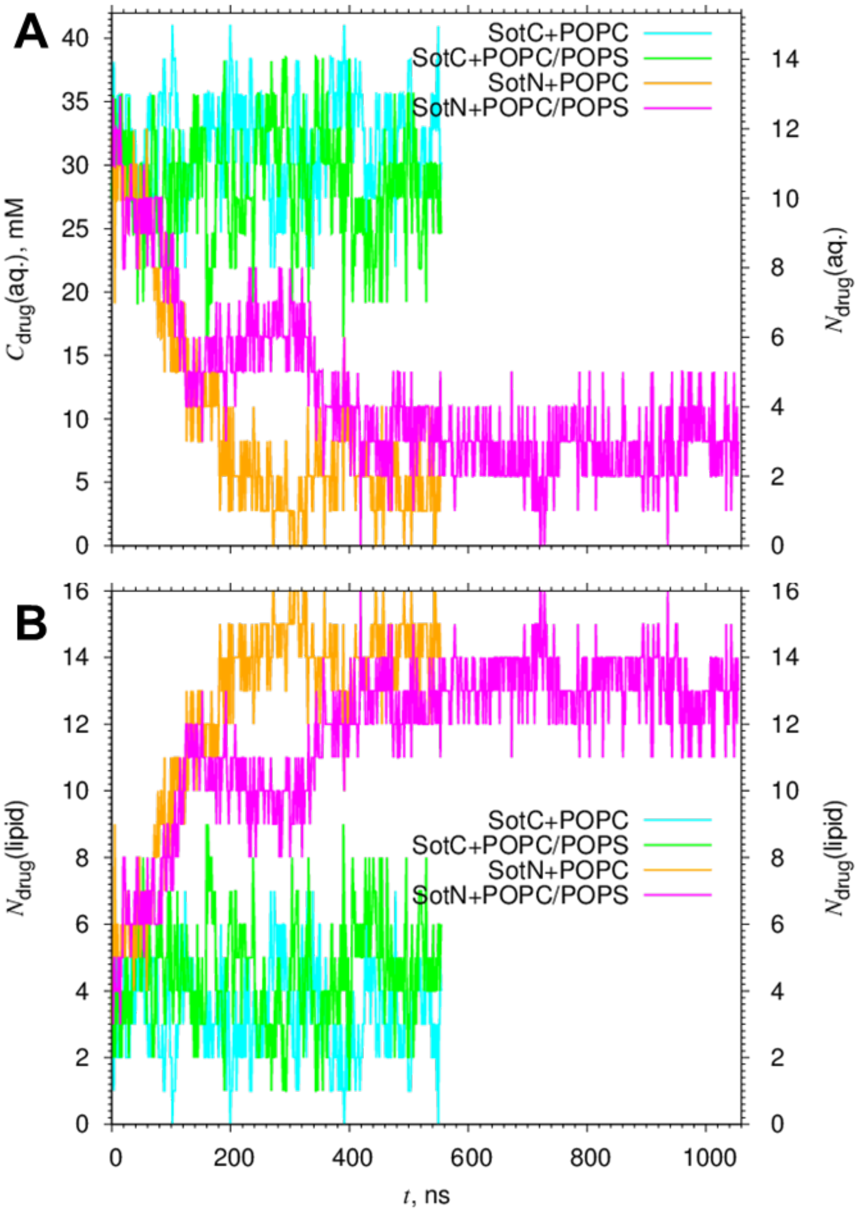

Figure S11. Time series of d-sotalol concentrations in bulk water and lipid membrane from unbiased MD simulations in POPC and POPC/POPS lipid membranes. (A) Number of drugs (SotC and SotN) in aqueous solution, N_{drug}(aq.), and corresponding concentration, C_{drug}(aq.), over the course of the trajectory with POPC and POPC/POPS simulations shown for comparison. **(B)** Number of drugs (SotC and SotN) in lipid membrane, N_{drug}(lipid), over the course of the trajectory with POPC and POPC/POPS simulations shown for comparison. Drug molecules were considered membrane bound when at least one of their non-hydrogen atoms was located within 3.5 Å from lipid non-hydrogen atoms.

Appendix S1. Neutral d-sotalol (SotN) optimized CHARMM force field topology and parameter files.

```
* Initial Topologies generated by
* CHARMM General Force Field (CGenFF) 3.0.1
*
36 1

! Penalties lower than 10 indicate the analogy is fair; penalties
between 10
! and 50 mean some basic validation is recommended; penalties higher
than
! 50 indicate poor analogy and mandate extensive
validation/optimization.

!==============================================================
! d-sotalol (0)
! Charge optimization by K. DeMarco 1/2017
!
! QM: {5.421 0.474 -1.472} (5.637 D)
! MM: {5.884 0.341 -1.024} (5.983 D)
! Ratio: 1.06
!
! Force Field ToolKit (ffTK)
! C.G. Mayne, J. Saam, K. Schulten, E. Tajkhorshid, J.C. Gumbart. J.
Comput. Chem. 2013, 34, 2757-2770.
!==============================================================

RESI SOT        0.000
GROUP              ! CHARGE   CH_PENALTY
ATOM S      SG3O2   0.426 !    0.000
ATOM H1     HGA1    0.090 !    0.800
ATOM O1     OG2P1  -0.381 !    0.000
ATOM O2     OG2P1  -0.381 !    0.000
ATOM N1     NG311  -0.071
ATOM N2     NG311  -0.456 !    0.000
ATOM C1     CG311   0.113
ATOM C2     CG321  -0.261
ATOM C3     CG311  -0.102
ATOM C4     CG2R61  0.006 !    5.682
ATOM C5     CG2R61 -0.110 !    2.149
ATOM C6     CG2R61 -0.110 !    2.149
ATOM C7     CG331  -0.279 !    4.872
ATOM C8     CG331  -0.279 !    4.872
ATOM C9     CG2R61  0.220 !    0.000
ATOM C10    CG2R61 -0.116 !    0.000
ATOM C11    CG2R61 -0.116 !    0.000
ATOM C12    CG331  -0.017 !    0.000
ATOM H2     HGPAM1  0.258 !    6.506
ATOM H3     HGP1    0.317 !    0.000
ATOM O3     OG311  -0.704
ATOM H4     HGA2    0.090 !    2.518
ATOM H5     HGA2    0.090 !    2.518
ATOM H6     HGA1    0.090 !    2.536
ATOM H7     HGR61   0.115 !    0.000
```

```
ATOM H8      HGR61   0.115 !    0.000
ATOM H9      HGA3    0.090 !    0.300
ATOM H10     HGA3    0.090 !    0.300
ATOM H11     HGA3    0.090 !    0.300
ATOM H12     HGA3    0.090 !    0.300
ATOM H13     HGA3    0.090 !    0.300
ATOM H14     HGA3    0.090 !    0.300
ATOM H15     HGR61   0.115 !    0.000
ATOM H16     HGR61   0.115 !    0.000
ATOM H17     HGA3    0.090 !    0.000
ATOM H18     HGA3    0.090 !    0.000
ATOM H19     HGA3    0.090 !    0.000
ATOM H20     HGP1    0.413 !    0.300

BOND S    O1
BOND S    O2
BOND S    N2
BOND S    C12
BOND H1   C1
BOND N1   C2
BOND N1   C3
BOND N2   C9
BOND C1   C2
BOND C1   C4
BOND C3   C7
BOND C3   C8
BOND C4   C5
BOND C4   C6
BOND C5   C10
BOND C6   C11
BOND C9   C10
BOND C9   C11
BOND N1   H2
BOND N2   H3
BOND C1   O3
BOND C2   H4
BOND C2   H5
BOND C3   H6
BOND C5   H7
BOND C6   H8
BOND C7   H9
BOND C7   H10
BOND C7   H11
BOND C8   H12
BOND C8   H13
BOND C8   H14
BOND C10  H15
BOND C11  H16
BOND C12  H17
BOND C12  H18
BOND C12  H19
BOND O3   H20

END
```

```
* Initial Parameters generated by analogy by
* CHARMM General Force Field (CGenFF) 3.0.1
*

* Penalties lower than 10 indicate the analogy is fair; penalties
between 10
* and 50 mean some basic validation is recommended; penalties higher
than
* 50 indicate poor analogy and mandate extensive
validation/optimization.


!=============================================================
! d-sotalol
! Parameter optimization by K. DeMarco 8/2016
!
! Force Field ToolKit (ffTK)
! C.G. Mayne, J. Saam, K. Schulten, E. Tajkhorshid, J.C. Gumbart. J.
Comput. Chem. 2013, 34, 2757-2770.
!=============================================================

BONDS
CG311  NG311   263.00     1.4740 ! ***** , from CG321 NG311,
penalty= 4

ANGLES
CG2R61 CG311  OG311    75.700   110.100 ! ***** , from CG2R61 CG321
OG311, penalty= 4
CG331  CG311  NG311    43.700   112.200 ! ***** , from CG331 CG321
NG311, penalty= 4
NG311  CG311  HGA1     32.400   109.500   50.00   2.13000 ! ***** ,
from NG311 CG321 HGA2, penalty= 4
CG311  CG321  NG311    43.700   112.200 ! ***** , from CG331 CG321
NG311, penalty= 1.5
CG311  NG311  HGPAM1   35.000   111.000 ! ***** , from CG321 NG311
HGPAM1, penalty= 0.6
CG311  NG311  CG321    71.657   115.117

DIHEDRALS
CG2R61 CG2R61 CG311  OG311      0.0000  2     0.00 ! ***** , from
CG2R61 CG2R61 CG321 OG311, penalty= 4
NG311  CG311  CG331  HGA3       0.1600  3     0.00 ! ***** , from
NG311 CG321 CG331 HGA3, penalty= 4
CG331  CG311  NG311  HGPAM1     0.1000  3     0.00 ! ***** , from
CG331 CG321 NG311 HGP1, penalty= 5.5
HGA1   CG311  NG311  HGPAM1     0.0500  3     0.00 ! ***** , from
HGA2 CG321 NG311 HGPAM1, penalty= 4
CG2R61 CG311  OG311  HGP1       2.1000  1     0.00 ! ***** , from
CG2R61 CG321 OG311 HGP1, penalty= 4
CG2R61 CG311  OG311  HGP1       1.4000  2     0.00 ! ***** , from
CG2R61 CG321 OG311 HGP1, penalty= 4
CG2R61 CG311  OG311  HGP1       0.7400  3     0.00 ! ***** , from
CG2R61 CG321 OG311 HGP1, penalty= 4
```

```
CG311  CG321  NG311  HGPAM1     0.1000  3     0.00 ! ***** , from
CG331 CG321 NG311 HGP1, penalty= 3
OG311  CG311  CG321  NG311   0.9490  3   180.00
CG311  CG321  NG311  CG311   0.7070  1     0.00
CG311  CG321  NG311  CG311   0.3200  2     0.00
CG311  CG321  NG311  CG311   2.4070  3   180.00
HGA1   CG311  NG311  CG321   0.7010  3   180.00
HGA1   CG311  CG321  NG311   0.7830  3     0.00
CG331  CG311  NG311  CG321   0.5570  1     0.00
CG331  CG311  NG311  CG321   0.0440  2   180.00
CG331  CG311  NG311  CG321   0.8730  3     0.00
CG2R61 CG311  CG321  NG311   1.3870  3     0.00
HGA2   CG321  NG311  CG311   1.5120  3     0.00

IMPROPERS

NONBONDED nbxmod  5 atom cdiel shift vatom vdistance vswitch -
cutnb 14.0 ctofnb 12.0 ctonnb 10.0 eps 1.0 e14fac 1.0 wmin 1.5

CG2R61  0.0  -0.070000  1.992400
CG311   0.0  -0.032000  2.000000  0.0  -0.010000  1.900000
CG321   0.0  -0.056000  2.010000  0.0  -0.010000  1.900000
CG331   0.0  -0.078000  2.050000  0.0  -0.010000  1.900000
HGA1    0.0  -0.045000  1.340000
HGA2    0.0  -0.035000  1.340000
HGA3    0.0  -0.024000  1.340000
HGP1    0.0  -0.046000  0.224500
HGPAM1  0.0  -0.009000  0.875000
HGR61   0.0  -0.030000  1.358200
NG311   0.0  -0.045000  2.000000
OG2P1   0.0  -0.120000  1.700000
OG311   0.0  -0.192100  1.765000
SG3O2   0.0  -0.470000  2.200000

END
```

<u>Appendix S2.</u> Cationic d-sotalol (SotC) optimized CHARMM force field topology and parameter files.

```
* Initial Topologies generated by
* CHARMM General Force Field (CGenFF) program version 1.0.0
*
36 1

! "penalty" is the highest penalty score of the associated
parameters.
! Penalties lower than 10 indicate the analogy is fair; penalties
between 10
! and 50 mean some basic validation is recommended; penalties higher
than
! 50 indicate poor analogy and mandate extensive
validation/optimization.

!==============================================================
! d-sotalol (+)
! Charge optimization by K. DeMarco 5/2017
!
! Dipole Moment (standard orientation basis):
! MM: {17.115 -3.287 -2.742} (17.642 D)
! QM: {15.074 -3.130 -1.762} (15.496 D)
! Ratio: 1.14
!
! Force Field ToolKit (ffTK)
! C.G. Mayne, J. Saam, K. Schulten, E. Tajkhorshid, J.C. Gumbart. J.
Comput. Chem. 2013, 34, 2757-2770.
!==============================================================

RESI SOT1         1.000
GROUP             ! CHARGE   CH_PENALTY
ATOM S      SG3O2   0.426 !    0.000
ATOM O1     OG2P1  -0.381 !    0.000
ATOM O2     OG2P1  -0.381 !    0.000
ATOM N1     NG3P2  -0.376 !    2.852
ATOM N2     NG311  -0.456 !    0.000
ATOM C1     CG311  -0.155
ATOM C2     CG324   0.295
ATOM C3     CG314   0.267 !    3.339
ATOM C4     CG2R61  0.190
ATOM C5     CG2R61 -0.110 !    6.048
ATOM C6     CG2R61 -0.110 !    6.048
ATOM C7     CG331  -0.265 !    2.437
ATOM C8     CG331  -0.265 !    2.437
ATOM C9     CG2R61  0.220 !    0.000
ATOM C10    CG2R61 -0.116 !    0.000
ATOM C11    CG2R61 -0.116 !    0.000
ATOM C12    CG331  -0.017 !    0.000
ATOM O3     OG311  -0.650 !    4.388
ATOM H1     HGP2    0.320 !    0.070
ATOM H2     HGP2    0.320 !    0.070
ATOM H3     HGP1    0.317 !    0.000
ATOM H4     HGA1    0.090 !    0.030
```

```
ATOM H5     HGA2    0.090 !    2.500
ATOM H6     HGA2    0.090 !    2.500
ATOM H7     HGA1    0.090 !    0.738
ATOM H8     HGR61   0.115 !    0.000
ATOM H9     HGR61   0.115 !    0.000
ATOM H10    HGA3    0.090 !    0.674
ATOM H11    HGA3    0.090 !    0.674
ATOM H12    HGA3    0.090 !    0.674
ATOM H13    HGA3    0.090 !    0.674
ATOM H14    HGA3    0.090 !    0.674
ATOM H15    HGA3    0.090 !    0.674
ATOM H16    HGR61   0.115 !    0.000
ATOM H17    HGR61   0.115 !    0.000
ATOM H18    HGA3    0.090 !    0.000
ATOM H19    HGA3    0.090 !    0.000
ATOM H20    HGA3    0.090 !    0.000
ATOM H21    HGP1    0.413 !    0.300

BOND S    O1
BOND S    O2
BOND S    N2
BOND S    C12
BOND N1   C2
BOND N1   C3
BOND N2   C9
BOND C1   C2
BOND C1   C4
BOND C3   C7
BOND C3   C8
BOND C4   C5
BOND C4   C6
BOND C5   C10
BOND C6   C11
BOND C9   C10
BOND C9   C11
BOND C1   O3
BOND N1   H1
BOND N1   H2
BOND N2   H3
BOND C1   H4
BOND C2   H5
BOND C2   H6
BOND C3   H7
BOND C5   H8
BOND C6   H9
BOND C7   H10
BOND C7   H11
BOND C7   H12
BOND C8   H13
BOND C8   H14
BOND C8   H15
BOND C10  H16
BOND C11  H17
BOND C12  H18
BOND C12  H19
```

```
BOND C12  H20
BOND O3   H21

END

* Initial Parameters generated by analogy by
* CHARMM General Force Field (CGenFF) program version 1.0.0
*

! Penalties lower than 10 indicate the analogy is fair; penalties
between 10
! and 50 mean some basic validation is recommended; penalties higher
than
! 50 indicate poor analogy and mandate extensive
validation/optimization.

!==============================================================
! d-sotalol (+)
! Parameter optimization by K. DeMarco 5/2017
! (No opt needed in this case)
!
! Force Field ToolKit (ffTK)
! C.G. Mayne, J. Saam, K. Schulten, E. Tajkhorshid, J.C. Gumbart. J.
Comput. Chem. 2013, 34, 2757-2770.
!==============================================================

BONDS

ANGLES
CG2R61 CG311  CG324    51.80    107.50 ! ***** , from CG2R61 CG321
CG314, penalty= 4.6
CG2R61 CG311  OG311    75.70    110.10 ! ***** , from CG2R61 CG321
OG311, penalty= 4
CG331  CG314  CG331    53.35    114.00    8.00   2.56100 ! ***** ,
from CG331 CG311 CG331, penalty= 1
CG331  CG314  NG3P2    67.70    110.00 ! ***** , from CG331 CG314
NG3P3, penalty= 0.9

DIHEDRALS
CG2R61 CG2R61 CG311  CG324      0.2300  2   180.00 ! ***** , from
CG2R61 CG2R61 CG321 CG314, penalty= 4.6
CG2R61 CG2R61 CG311  OG311      0.0000  2     0.00 ! ***** , from
CG2R61 CG2R61 CG321 OG311, penalty= 4
CG2R61 CG311  CG324  NG3P2      0.2000  3     0.00 ! ***** , from
NG3P3 CG314 CG321 CG2R61, penalty= 8.9
CG2R61 CG311  CG324  HGA2       0.0000  3     0.00 ! ***** , from
CG2R61 CG311 CG321 HGA2, penalty= 1
OG311  CG311  CG324  NG3P2      0.1950  3     0.00 ! ***** , from
OG311 CG311 CG324 NG3P1, penalty= 0.6
CG2R61 CG311  OG311  HGP1       2.1000  1     0.00 ! ***** , from
CG2R61 CG321 OG311 HGP1, penalty= 4
CG2R61 CG311  OG311  HGP1       1.4000  2     0.00 ! ***** , from
CG2R61 CG321 OG311 HGP1, penalty= 4
CG2R61 CG311  OG311  HGP1       0.7400  3     0.00 ! ***** , from
CG2R61 CG321 OG311 HGP1, penalty= 4
```

```
CG331  CG314  CG331  HGA3       0.1950  3     0.00 ! ***** , from
CG331 CG311 CG331 HGA3, penalty= 1
NG3P2  CG314  CG331  HGA3       0.2000  3     0.00 ! ***** , from
NG3P3 CG314 CG331 HGA3, penalty= 0.9
CG331  CG314  NG3P2  CG324      0.1000  3     0.00 ! ***** , from
CG321 CG314 NG3P2 CG324, penalty= 0.9
CG331  CG314  NG3P2  HGP2       0.1000  3     0.00 ! ***** , from
CG321 CG314 NG3P2 HGP2, penalty= 0.9
CG311  CG324  NG3P2  CG314      0.4000  1     0.00 ! ***** , from
CG311 CG324 NG3P2 CG324, penalty= 0.6
CG311  CG324  NG3P2  CG314      0.2500  2     0.00 ! ***** , from
CG311 CG324 NG3P2 CG324, penalty= 0.6
CG311  CG324  NG3P2  CG314      0.6000  3     0.00 ! ***** , from
CG311 CG324 NG3P2 CG324, penalty= 0.6

IMPROPERS

NONBONDED nbxmod  5 atom cdiel shift vatom vdistance vswitch -
cutnb 14.0 ctofnb 12.0 ctonnb 10.0 eps 1.0 e14fac 1.0 wmin 1.5

CG2R61  0.0  -0.070000  1.992400
CG311   0.0  -0.032000  2.000000  0.0  -0.010000  1.900000
CG314   0.0  -0.031000  2.165000  0.0  -0.010000  1.900000
CG324   0.0  -0.055000  2.175000  0.0  -0.010000  1.900000
CG331   0.0  -0.078000  2.050000  0.0  -0.010000  1.900000
HGA1    0.0  -0.045000  1.340000
HGA2    0.0  -0.035000  1.340000
HGA3    0.0  -0.024000  1.340000
HGP1    0.0  -0.046000  0.224500
HGP2    0.0  -0.046000  0.224500
HGR61   0.0  -0.030000  1.358200
NG311   0.0  -0.045000  2.000000
NG3P2   0.0  -0.200000  1.850000
OG2P1   0.0  -0.120000  1.700000
OG311   0.0  -0.192100  1.765000
SG3O2   0.0  -0.350000  2.000000
END
```
